# Peripheral blood-derived monocytes show neuronal properties and integration in immune-deficient rd1 mouse model upon phenotypic differentiation and induction with retinal growth factors

**DOI:** 10.1186/s13287-020-01925-y

**Published:** 2020-09-23

**Authors:** Alaknanda Mishra, K. Varsha Mohan, Perumal Nagarajan, Srikanth Iyer, Ashwani Kesarwani, Madhu Nath, Laxmi Moksha, Jashdeep Bhattacharjee, Barun Das, Kshama Jain, Parul Sahu, Prakriti Sinha, T. Velapandian, Pramod Upadhyay

**Affiliations:** 1 National Institute of Immunology, Aruna Asaf Ali Marg, New Delhi, 110067 India; 2 Department of Ocular Pharmacology, Dr. Rajendra Prasad Centre for Ophthalmic Sciences, All India Institute of Medical Sciences, New Delhi, 110029 India

**Keywords:** Retinitis pigmentosa, Human peripheral blood monocytes, Retinal neuron-like cells, Visual activity, rd1 mouse model

## Abstract

**Background:**

Cell therapy is one of the most promising therapeutic interventions for retinitis pigmentosa. In the current study, we aimed to assess if peripheral blood-derived monocytes which are highly abundant and accessible could be utilized as a potential candidate for phenotypic differentiation into neuron-like cells.

**Methods:**

The peripheral blood-derived monocytes were reconditioned phenotypically using extrinsic growth factors to induce pluripotency and proliferation. The reconditioned monocytes (RM) were further incubated with a cocktail of growth factors involved in retinal development and growth to induce retinal neuron-like properties. These cells, termed as retinal neuron-like cells (RNLCs) were characterized for their morphological, molecular and functional behaviour in vitro and in vivo.

**Results:**

The monocytes de-differentiated in vitro and acquired pluripotency with the expression of prominent stem cell markers. Treatment of RM with retinal growth factors led to an upregulation of neuronal and retinal lineage markers and downregulation of myeloid markers. These cells show morphological alterations resembling retinal neuron-like cells and expressed photoreceptor (PR) markers. The induced RNLCs also exhibited relative membrane potential change upon light exposure suggesting that they have gained some neuronal characteristics. Further studies showed that RNLCs could also integrate in an immune-deficient retinitis pigmentosa mouse model NOD.SCID-rd1 upon sub-retinal transplantation. The RNLCs engrafted in the inner nuclear layer (INL) and ganglion cell layer (GCL) of the RP afflicted retina. Mice transplanted with RNLCs showed improvement in depth perception, exploratory behaviour and the optokinetic response.

**Conclusions:**

This *proof-of-concept* study demonstrates that reconditioned monocytes can be induced to acquire retinal neuron-like properties through differentiation using a defined growth media and can be a potential candidate for cell therapy-based interventions and disease modelling for ocular diseases.

## Background

Retinitis pigmentosa (RP) is a progressive irreversible retinal degenerative disease which causes partial or complete blindness [1, 2]. This phenomenon predominantly affects rod photoreceptors, and subsequently, other retinal cells are also affected [3]. Mutations in the gene PDE6B encoding for the β-subunits of phosphodiesterase 6 enzyme cause autosomal recessive RP. Hence, the mechanisms that initiate accumulation of cGMP and calcium are thought to cause rod-cone degeneration in Pde6b−/− RP patients [4].

Currently, the condition is incurable, and the treatment modules offer only symptomatic relief. Some of the treatment approaches include neuroprotective strategies [5, 6], gene therapy [5], prosthetic devices [7, 8], and stem cell therapies [2, 9, 10]. Reports have shown that cell therapy could not only replenish the degenerated retina but the transplanted cells could also form synaptic connections with the preserved population of cells [10]. Besides, cell-based therapy can benefit multi-loci genetic mutations by possibly recreating the natural anatomy and circuitry of retina.

However, it is difficult to find an accessible and abundant source of cells using minimally invasive techniques for this purpose. Patient-specific iPSCs are currently under intense scrutiny for their unpredictable genomic instability [11] while ESCs have various ethical issue concerns [12]. The patient-specific retinal somatic tissue obtained from cadavers or after retinal biopsies obtained during intraocular retinectomy are usually inadequate for disease modelling or drug screening.

The monocytes comprise 5–8% of blood, thus yielding a larger endpoint population. Monocytes are promising target for differentiation into neuronal lineages due to established fact that (i) there is no need for viral insertion (or other non-integrative techniques) that is known to cause methylation abnormalities as well as mutations [13, 14], (ii) they have a shorter time of differentiation from somatic cell to neuronal-like cell when compared to iPSC [15], and (iii) preliminary results indicating monocytes deliver reproducibility with serial samples from the same individual [15] which continues to be a challenge with iPSCs [16–18].

Therefore in our present study, we aimed to assess the potential of monocytes obtained from peripheral blood to differentiate without genetic modification, to stem cell-like cells as reported earlier [19]. Thereafter, we induced the reconditioned monocytes with extrinsic factors involved in retinal differentiation towards a state mimicking retinal neuron-like cells. The term reconditioning/differentiation in this study refers to phenotypic alteration and/or induction of cells without genetic manipulation. RNLCs were studied for molecular, phenotypic and functional properties in vitro followed by transplantation in an immune-deficient rd1 mouse model previously developed in our lab [20] to test their integration in vivo. We also checked if these induced RNLCs could gain retinal neuron-like function when aided by an ocular microenvironment upon transplantation.

## Materials and methods

### Ethical clearance for use of human blood samples and animal experiments

Ethical clearance was obtained from Institutional Human Ethics Committee of National Institute of Immunology, New Delhi, for procuring buffy coat of healthy blood samples (IHEC#85/14). Approval to perform animal experiments was obtained from Institutional Animal Ethics Committee of National Institute of Immunology, New Delhi (IAEC#334/14).

### Animal housing and breeding

All the mice required during different experiments (NOD.CB17-Prkdcscid/J (NOD SCID), CBA/J and BALB/cByJ (BALB/c) were procured from the Jackson Laboratory, USA while the NOD-SCID-rd1 mouse model was developed in-house as reported previously [20]. The animals received ad libitum access to acidified autoclaved water and food. The temperature and humidity of the housing room was maintained at 21–23 °C and 40–60% respectively. Animals were kept at a 14-h light to 10-h dark cycle. Animals were randomly assigned in each group, and experiments were repeated at least twice. All animal experiments and reporting adhere to the ARRIVE guidelines [21].

### Peripheral blood mononuclear cell (PBMC) isolation

The buffy coat of the blood donated by healthy volunteers was obtained from the blood bank of All India Institute of Medical Sciences (AIIMS), New Delhi. The samples were negative for human immunodeficiency virus (HIV), hepatitis B virus (HBV) and hepatitis C virus (HCV). PBMCs were isolated from buffy coat by density centrifugation technique using Hisep ficoll (HiMedia, India; 1.077 g/cm^3^) and washed with PBS before plating for monocyte enrichment. The isolated PBMCs isolated from the buffy coat were plated for overnight in IMDM supplemented with 5% fetal bovine serum (FBS) (Gibco, USA) in T-150 flasks and incubated at 37 °C and 5% CO_2_. The non-adherent cells were discarded, and the adherent cells were washed twice with PBS to ensure removal of any non-adherent cells. The adherent cells were isolated by trypsinization (0.25% trypsin) and centrifuged at 300*g* for 10 min at 4 °C. The pellet obtained was re-suspended in incomplete IMDM media, and cells were counted using trypan blue. To obtain highly pure monocyte cell population, the adherent cells were sorted for CD14+ cells using FACS ARIA II (BD Biosciences, USA).

### Viability characterization

The adherent cells were checked for viability by annexin-A5 and propidium iodide (PI) staining according to the manufacturer’s instruction (BD Biosciences, USA). Briefly, 1 × 10^6^ cells were diluted in 1X annexin-binding buffer and 5 μL FITC annexin V (Component A) and 1 μL PI working solution (100 μg/mL) was added to 100 μL cell suspension. The cells were incubated at room temperature for 15 min, and the reaction was stopped by adding 400 μL of 1X annexin-binding buffer. After mixing it gently, the samples were kept on ice. The stained samples were analysed immediately by flow cytometry (FACS Verse), and data was analysed using FlowJo®.

### Differentiation of monocytes into RNLCs

1 × 10^6^ cells/ well were seeded in human fibronectin coated (5 μg/cm^2^) 12-well plates supplemented with culture media prepared in IMDM supplemented with IL3 (4 ng/ml), MCSF (5 ng/ml), 2-ME (140 μM) and embryonic stem cell grade serum (0.5%). The obtained reconditioned monocytes (RM) obtained post 6 days of cell culture and were induced towards neuronal lineage by culturing them in DMEM/F-12 supplemented with b-FGF (20 ng/ml), Retinoic acid (0.1 μg/ml), EGF (20 ng/ml), IGF-1 (20 ng/ml), SCF (20 ng/ml), Taurine (100 μM), B27 supplement (2%), ESC serum (0.5%), MEM-NEAA (1x) and antibiotics (1%) for 8 days. Media was changed every 3 days during the culture tenure. Manufacturer’s information for growth factors and supplements is provided in Table 8.

### Morphological studies

The morphology of the cells was imaged using a phase contrast microscope at × 10 and × 20 resolution for different stages of culture tenure. The morphology of cells at each stage was reconfirmed by scanning electron microscopy (Zeiss, at 20 kV and 4.97Kx magnification). The method for SEM analysis has been detailed in Supplementary data file.

### MTT assay

Cells were plated at 5 × 10^5^ cells/well concentration and incubated overnight for adherence. DMEM-F12 media containing 0.5 mg/ml MTT (3-(4,5-dimethylthiazol-2-yl)-2,5-diphenyl tetrazolium bromide) was added to the cells and incubated for 3 h at 37 °C. The blue formazan crystals were dissolved in dimethyl sulphoxide (DMSO), and absorbance was measured at 570 nm.

### qPCR analysis

The cells were harvested by trypsinization (0.25% trypsin) and RNA was isolated using TRI Reagent (Sigma, USA) as per the manufacturer’s protocol. RNA pellet obtained post spin was then washed twice with 70% alcohol, air dried and resuspended in nuclease-free water. cDNA was prepared using cDNA synthesis kit (Biorad, USA) according to the manufacturer’s protocol, and real-time PCR was performed using DyNAmo Flash SYBRGreen (Thermo Fisher Scientific, USA) on the Mastercycler® Realplex platform (Eppendorf, Germany). Primers are tabulated in supplementary data file Table 2–7.

### Flow cytometry

The adherent cells (1 × 10^6^ cells) were harvested and resuspended in 100 μl of a cocktail of anti-human cell surface marker antibodies at a dilution of 1:200 in PBS and mixed gently. The cells were incubated at 4 °C for 40 min in dark. The stained cells were washed with 1X PBS after incubation and analysed immediately.

For evaluation of intracellular markers, fresh fixation/permeabilization solution was prepared by diluting the fix/perm concentrate with diluent at a ratio of 1:3 (BD bioscience, USA). One millilitre Fix/Perm solution was added to each sample, incubated for 1 h protected from light and centrifuged at 300*g* for 5 min at RT. The cells were washed and blocked with 2% FBS in permeabilization buffer for 15 min. The cells were washed again, and appropriate dilutions of antibodies were added and incubated for 30 min at RT in dark. The samples were washed and incubated in 100 μl of secondary antibody for 20 min at RT. Appropriate single-colour controls and secondary antibody controls were also prepared. The samples were washed and run on BD FACSVerse™. The data was analysed using FACS Diva software. The antibodies are tabulated in Supplementary data file Table 1.

### 5-Ethynyl-2′-deoxyuridine (EDU) proliferation assay

EDU (5-ethynyl-2′-deoxyuridine) cell proliferation assay was performed using Click-It EDU-Alexa Fluor 488 assay kit as per the manufacturer’s instruction (Thermo fisher scientific, USA). Briefly, the cells were plated at an optimum density and EDU was added to the culture medium at a concentration of 10 μM, mixed well and incubated for 1–2 h. The EDU-treated cells were washed with 3 ml of 1% BSA in PBS and cells were pelleted. One hundred microlitres of fixative (Component D) was added to the pellet, vortexed and incubated for 15 min at RT in dark followed by a wash with 1% BSA in PBS and centrifuged to obtain a pellet. The cell pellet was resuspended in 1X Click-It permeabilization buffer and incubated for 15 min at RT. The permeabilized cells were washed with 3 ml of 1X Click IT saponin-based wash reagent, centrifuged and resuspended in PBS. The samples were run in FACS Verse and analysed by Flowjo 10 software for Alexa Fluor 488 positive cells.

### Western blotting

The isolated cells were lysed in RIPA lysis buffer and centrifuged, the supernatant was collected and protein estimation using BCA kit (G-Biosciences, USA) was performed according to the manufacturer’s instructions. The samples were run on 10% SDS-Polyacrylamide gel and transferred to a PVDF membrane. The membrane was incubated overnight in primary antibody at 4 °C (1: 1000) followed by 3X washes for 10 min each and further incubated for 2 h in anti-mouse/rabbit secondary antibody (1:1000) conjugated with HRP. After 2 washes with PBST, the blot was treated with ECL substrate mixture (Thermo Fisher Scientific, USA) for 5 min in dark and developed using a ChemiDoc™ (Biorad, USA). The antibodies utilized are tabulated in Supplementary data file Table 1.

### Immunocytochemistry (ICC)

The cells were fixed with 4% PFA for 30 min at 4 °C in dark. The fixed cells were permeabilized for 45 min using permeabilization buffer containing 0.1% saponin, followed by washing and blocking with 5% BSA for 45 min at RT. The cells were washed with wash buffer and incubated in primary antibodies (Supplementary data file Table 1) diluted at proper optimized concentrations for an hour at RT or overnight at 4 °C. After 3 washes with PBS, the cells were incubated with a fluorochrome-labelled secondary antibody for 40–45 min at RT, washed thrice with PBS and counterstained with DAPI (dilution 1:500; Concentration: 1 mg/ml) for 5 min at RT. The cells were mounted with coverslips using Vectashield®. Representative images were captured using a fluorescent microscope (× 40) or a confocal microscope (LSM Zeiss 510) at × 63.

### Potentiometric dye-based study of relative membrane potential changes in photopic and scotopic conditions

We prepared 5 μM DiBAC_4_(3) with 20% Pluronic® F-127 in 1 ml culture medium (Stock concentration of dye was 100 μM). The cells were loaded with 5 μl of dye solution and incubated in dark for 5 min. The control samples included untreated control to which only DMSO was added, hyperpolarization control to which 10 μl of 1 mM valinomycin was added and depolarization control to which 10 μl of 1 mM gramicidin was added. Auto-fluorescence was compensated using an only dye-treated control. The samples incubated in light and dark conditions were used to observe membrane potential changes in light/dim conditions. The samples were analysed by flow cytometry (FACSVerse™) and fluorescence was measured.

### Carboxyfluorescein succinimidyl ester (CFSE) labelling of RNLC prior to transplantation

CFSE was used for labelling the RNLCs before transplantation. The cells were isolated, washed with 1X PBS and enumerated. A working concentration of 20 μM of CFSE in HBSS was prepared and added to the cells. The cells were incubated with CFSE for 15 min at 37 °C. The labelling reaction was quenched by adding 5 volumes of cold complete media and incubated on ice for 5 min, protected from light. The stained cells were centrifuged at 300*g* for 5 min at RT and washed twice with PBS before use.

### Cell engraftment analysis

The engraftment efficiency of transplanted cells in RP mouse model was evaluated by fluorescent imaging and qPCR analysis. qPCR was performed using a human GAPDH-specific TaqMan probe. The details of qPCR methodology, primers and setup conditions are given in the Supplementary data file.

### Fluorescent in situ hybridization (FISH)

The animals were euthanized, and the eyes were enucleated, washed and fixed in freshly prepared 4% PFA. The tissue samples were embedded in OCT (optimum cutting temperature) medium and cryo-sectioned to obtain 5 μm sections on L-poly-lysine coated slides.

The biotin labelled DNA probe was added to the tissue section, covered with a cover glass and sealed using rubber cement. The slides were hybridized at 37 °C for 16–24 h. The cover glass was then removed and the slides were washed. After washing, the slides were incubated with secondary antibody (conjugated with Streptavidin Alexa Fluor 594) staining followed by counterstaining with DAPI. The slides were mounted with coverslip and visualized and imaged in confocal microscope (Zeiss LSM) at × 63 magnification. Detailed method is provided in the Supplementary data file.

### c-GMP analysis

c-GMP concentration was measured using DetectX® cGMP colorimetric kit (Arbor Assays, USA, Cat: K020-H1) from serum and eye lysate samples of non-transplanted and transplanted animals as per the manufacturer’s protocol. Briefly, we prepared standards from stock c-GMP solution (640 pmol/mL) by serial dilution at 20-fold dilution with each step to obtain concentrations ranging from 32 to 0.5 pM/ml. The standards were used within 1 h of preparation. Samples were prepared by diluting them appropriately in assay diluent. Fifty microlitres of plate primer was added in the wells followed by adding 75 μl of sample diluent into the non-specific binding (NSB) wells. Fifty microlitres of sample diluent was added into the maximum binding (B0) wells. Fifty microlitres of samples or standards was dispensed into the wells, and 25 μl of DetectX® cGMP conjugate was added to each well followed by addition of 25 μl of DetectX® cGMP antibody except NSB wells. The plate was gently tapped from the sides to ensure adequate mixing of the reagents. The plate was covered with plate sealer and kept at shaker for 2 h at RT. The wells were aspirated, washed four times with 300 μl wash buffer and tapped dry. Further, 100 ul of TMB (Tetramethylbenzidine) substrate was added to each well and incubated at RT for 30 min. The reaction was quenched by adding 50 μl stop solution. The absorbance maxima were read at 450 nm, and standard curve was plotted. cGMP concentration was calculated.

### Transplantation via trans-corneal subretinal injection

NOD.SCID-rd1 mice (4–6 weeks) of any gender (2:1 male to female ratio) were used for the experiments. The cornea was topically anaesthetized by one drop of proparacaine hydrochloride ophthalmic solution (0.5%). A single drop of tropicamide ophthalmic solution (1%) was used to dilate pupil. The animal was anaesthetized using ketamine (80-100 mg/kg) and xylazine (10 mg/kg) intraperitoneally. After anaesthetizing, the mouse was placed in lateral recumbence with the eye to be injected facing up. The skin was retracted towards the body causing the eye to protrude. The needle bevel was inserted up into the corneal of the eye at a 45 degree angle to relieve ocular pressure. The cell suspension of 1 million RNLCs was injected into the sub-retinal region. After injection, ophthalmic Tobrex ointment/povidine-iodine (5% solution) was applied along with antibiotic solution on the mouse eye to prevent infection. The animals were placed in IVC cages and monitored for viability and signs of eye infections like redness, keratitis and puffiness. We injected only one eye for IHC, FISH and cell engraftment quantification while both eyes were injected for behavioural analysis. The non-transplanted animals were used as negative control in behavioural studies.

### Immunohistochemistry

Whole eye was enucleated from the animals after euthanasia by cervical dislocation. The tissue was washed in PBS once to remove any blood and was immersed in 4% PFA for 2 h at 4 °C. The tissue was washed with PBS once to remove PFA and transferred to 30% sucrose prepared in PBS and kept overnight. The eye was embedded in OCT medium, and 8-μm-thick cryo-sections were obtained on poly-l-lysine-coated slides. The tissue sections were fixed using 4% PFA for 15 min at RT followed by 3X washes with PBS for 5 min each. The sections were then permeabilized for 45 min at RT with saponin-based buffer and blocked in 5% BSA solution for 30–45 min at RT followed by 2 washes for 10 min each. Primary antibodies were added to sections at appropriate dilutions and incubated overnight followed by secondary antibody incubation for 40 min (1:500 dilution). The nuclei were stained by DAPI at a dilution of 1:500 for 5 min, and slides were washed with PBS before mounting the slides using Vectashield. The representative confocal images were taken at × 63 magnification using a system incorporated in the microscope (Zeiss LSM Version 4.2.0.121). Details of primary antibodies used can be found in Supplementary data file Table 1.

### Behavioural analysis

Different groups of animals were analysed for behavioural changes before and after transplantation of RNLCs by visual cliff test, light/dark latency test and optokinetic response test. Detailed methods are given in the Supplementary data file.

### Statistical analysis

All statistical analysis was performed using one way or two way ANOVA post hoc Bonferroni tests. Prior to analysis, data were tested for normality and equal variance using descriptive statistics to ensure that ANOVA assumptions were met. Multi-parametric comparisons between groups were tested with two tailed *t* tests. *P* values were corrected for multiple testing if multiple comparisons were performed. *P* values less than 0.05 were considered significant.

## Results

### Reconditioned monocytes exhibit pluripotency and proliferation

**Fig. 1:**
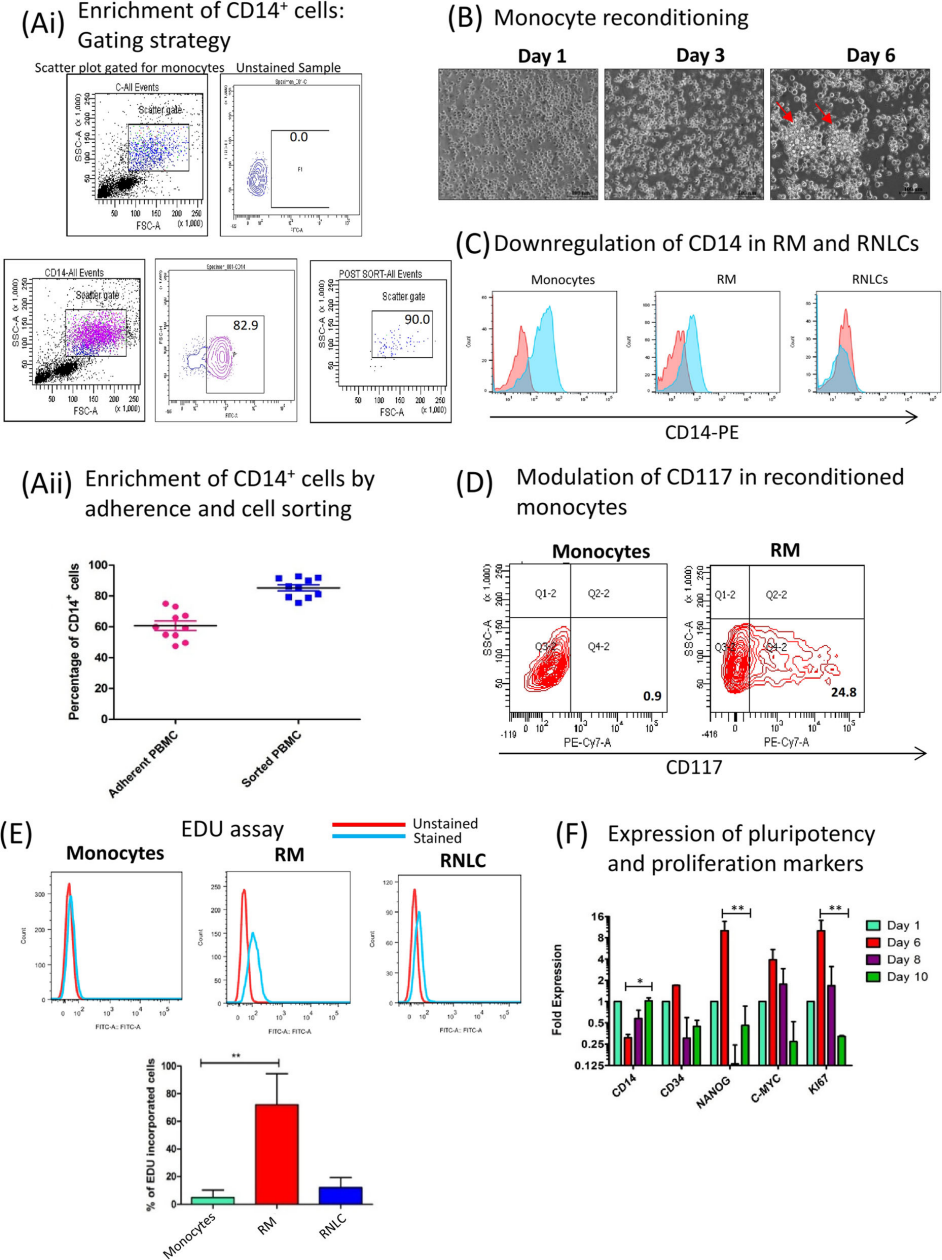
Isolation, recondition and characterization of PBMC derived monocytes into RM. **a** (i) PBMCs were isolated by density gradient centrifugation method, wherein the buffy coat was diluted in RPMI-1640 media (1:3) which was layered over ficoll. The obtained PBMCs were plated overnight. Thereafter, the non-adherent cells were discarded and the adherent monocyte cells were isolated by trypsinization. The monocytes isolated based on adherence showed reduced purity (65.9%) as seen by flow cytometric analysis of CD14+ cells gated in side scatter plot. **a** (ii) Graphical representation of adherent and sorted CD14^+^ monocyte population showing higher percentage of CD14^+^ cells in sorted culture than adherent culture. Around 54.5 ± 9.39% cells of adherent PBMCs stained positive for CD14 and 79.5 ± 6.24% cells in FACS sorted population were CD14 positive, (*n* = 10). **b** Bright field images at × 20 resolution showing kinetics of monocyte recondition and morphology alteration. The small round monocytes at day 1 formed colonies and became large and rounded at day 6 (known as reconditioned monocytes or RM). **c** Flow cytometric analysis of CD14, a monocyte-specific marker, was downregulated in RM and RNLCs and **d** CD117 was upregulated in RM as compared to monocytes suggesting molecular and phenotypic changes. **e** 5-Ethynyl-2′-deoxyuridine (EDU) assay was performed to analyse the proportion of proliferative cells. RM showed significant proliferation capacity as against negligible proliferation in monocytes and sparse proliferation in RNLCs. A graphical representation of the EDU analysis is also shown (*n* = 5). **f** Kinetic study through qPCR was performed to analyse the pluripotency and proliferation in RM which indicated that RM had a transient expression marker responsible for pluripotency (CD34, NANOG, c-myc) and proliferation markers (KI67) and the stem cell-like properties peaked at day 6 which declined further (*n* = 5). *p < 0.05; **p < 0.01; ***p < 0.001

The CD14^+^ monocytes obtained from PBMCs by enrichment on plastic tissue culture flasks and flow cytometric sorting exhibited a purity of 54.5 ± 9.39% and 75.5 ± 6.24% respectively (Fig. 1a (i, ii)). These cells were differentiated into reconditioned monocytes (RM) by culturing them in media consisting IL-3, MCSF and β-ME in a low serum environment as reported earlier [17]. The monocytes upon de-differentiation became larger in size, rounded and formed colonies by day 6 in culture (Fig. 1b). Flow cytometric and qPCR studies suggested a downregulation of CD14 expression in RM (Fig. 1c) and 25% increase in the expression of CD117 compared to 0.9% in day 1 monocytes (Fig. 1d). RM cells (72.06 ± 18.34%) incorporated AF-488 tagged EDU, a thymidine analogue suggesting their active proliferation stage (Fig. 1e) which is absent in terminally differentiated monocytes. We also observed that the expression of Ki67 (*p* < 0.001), CD34, Nanog (*p* < 0.001) and c-myc is highest at day 6 followed by a decrease in their expression by day 10, suggesting that proliferative nature of RM is transient. CD14 expression (*p* < 0.05) also decreases at day 6 during de-differentiation but is elevated back at day 10 in de-differentiation culture (Fig. 1f).

**Fig. 2:**
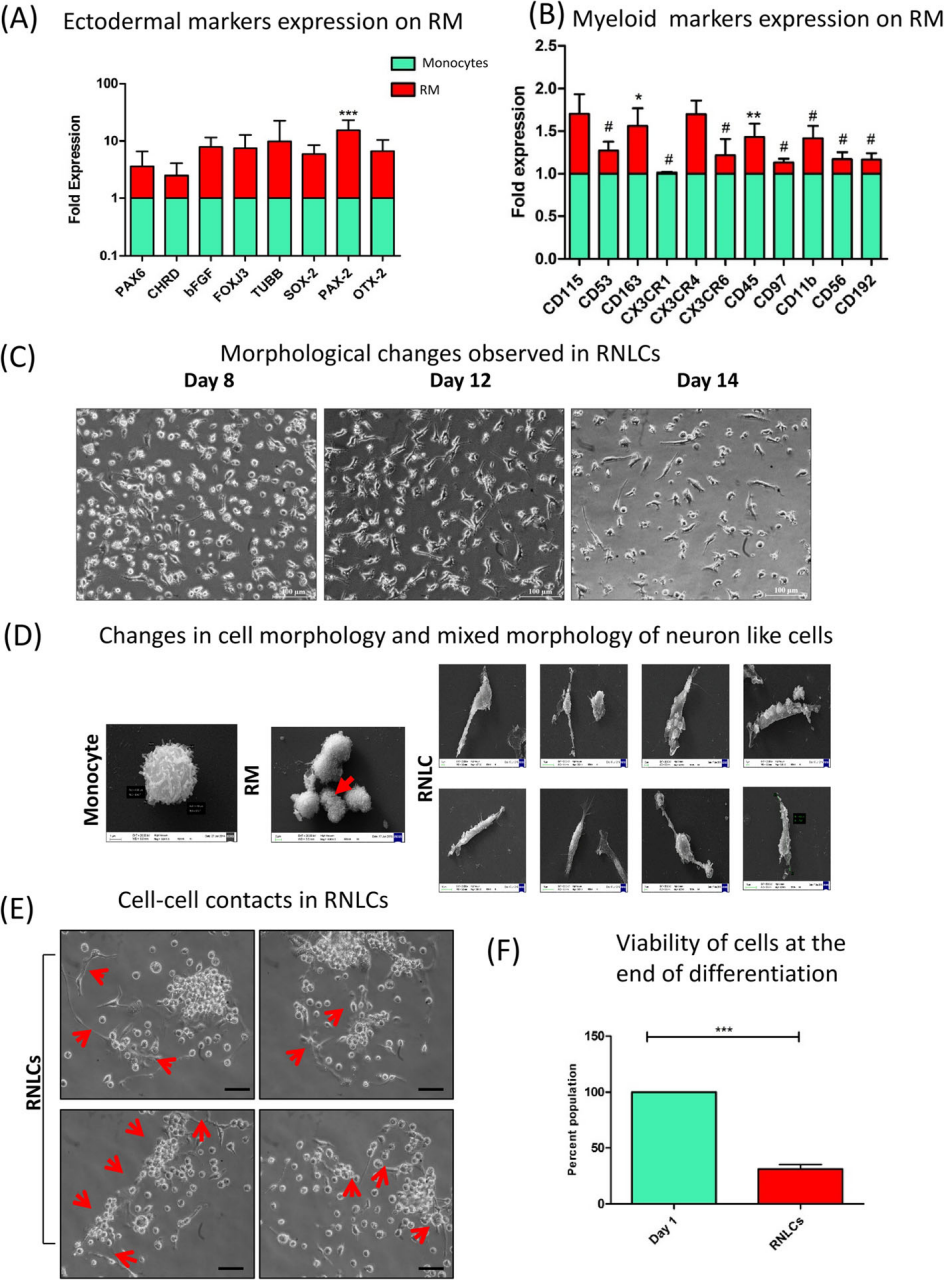
Characterization of lineage switch in reconditioned monocytes (RM) and their in vitro induction into retinal neuron-like cells (RNLCs). **a** Ectodermal markers were upregulated as compared to day 1 monocytes. **b** RM also contributed to express myeloid lineage markers (*n* = 4). **c** Morphology changes observed at × 20 upon induction with extrinsic retinal differentiation growth factors, where round and colony-forming RM formed long, elongated, round and axonal cells which we termed as retinal neuron-like cells (RNLCs) at day 14. **d** SEM images depicting a typical monocyte which further changes to dividing RM (indicated by red arrow) and eventually to RNLCs exhibiting mixed morphology of neuron-like cells. **e** The RNLCs also exhibited cell-cell contacts as indicated by red arrows**. f** Approximately 40% cells of the initial PBMCs were live and viable after induction at the culture endpoint as indicated by MTT assay

We analysed the expression of myeloid and ectodermal markers in RM as compared to monocytes. Monocyte is a cell type of myeloid cellular origin, and therefore, we wanted to determine if the reconditioned monocytes differed in their lineage marker expression. We found that RM exhibited significant elevation in *Pax2* expression while there was no statistical difference in levels of *PAX6*, *OTX2*, *TUBB*, *BFGF*, *CHRD*, *FOXJ3* and *SOX2* (Fig. 2a). The expression of myeloid lineage genes including CD53, CX3CR1, CX3CR6, CD45, CD97, CD11b, CD56 and CD92 was significantly downregulated in RM (Fig. 2b).

### Induction with retinal cell differentiation-related extrinsic growth factors causes morphological alteration and retinal marker expression in RM

Subsequently, RM were cultured in a cocktail of growth factors (IGF-1, bFGF, SCF, RA, EGF, B-27 supplement, MEM-NEAA, ITS (Insulin-Transferrin-Selenium) supplement and 0.5% ESC grade serum) for 8 days to induce them towards a retinal neuron lineage. The RNLCs, obtained at the end of culture, were composite and exhibited a myriad of morphologies as confirmed by bright field images and SEM analysis (Fig. 2c, d). RNLCs were also capable of cell-cell contacts in vitro through cell extensions (Fig. 2e). The yield of RNLCs obtained at the end of culture term was around 40.5 ± 11.03% as indicated by MTT analysis (Fig. 2f).

**Fig. 3:**
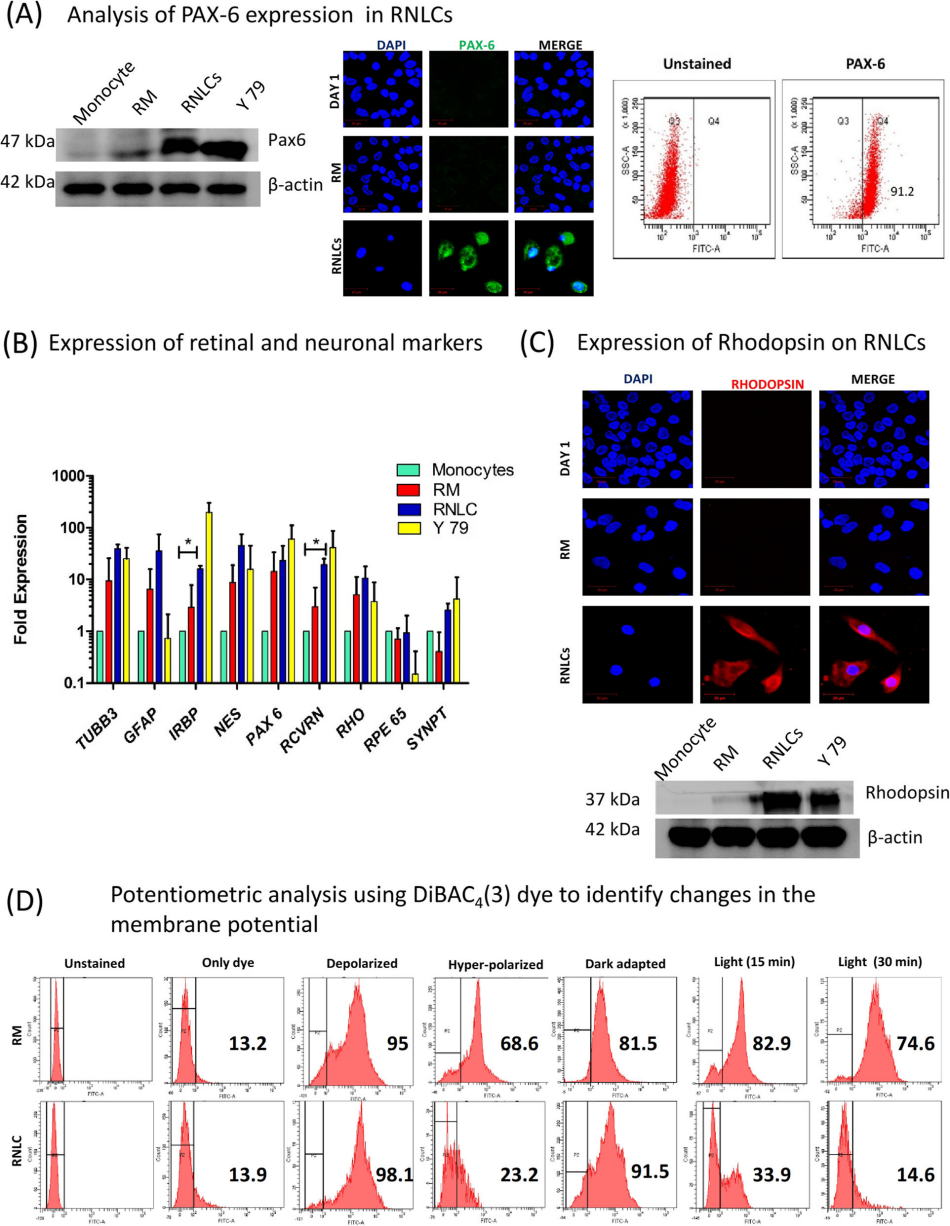
Characterization of ectodermal and neuronal properties of in vitro induced RNLCs. **a** PAX-6, an ectodermal and a pan-neuronal/retinal markers, showed expression in RNLCs as shown by both western blotting and ICC, and flow cytometric analysis suggested that around 90% of the RNLCs were PAX-6 positive. The expression was analysed by flow cytometry, western blotting and ICC. **b** qPCR analysis of retinal markers in RNLCs, RM and retinoblastoma cell line Y79 (positive control) (*n* = 5). *p < 0.05; **p < 0.01. **c** RNLCs expressed Rhodopsin indicating that these cells had an induced retinal neuron-like properties. The images were captured at × 63 magnification. The western blot analysis also confirmed that RNLCs expressed Rhodopsin after retinal growth factor induction. **d** Potentiometric analysis for the changes in membrane potential during light and dark conditions were analysed by using DiBAC4(3) dye. An only dye-treated control was an auto-fluorescence control. Cells treated with valinomycin and gramicidin-D were used as depolarization (DP) and hyperpolarization (HP) controls. The changes in fluorescent intensity were monitored when the cells where dark-adapted and light-exposed for 15 min and 30 min respectively. It was observed that RM did not exhibit any noticeable change between dark- (81.5%) and light-exposed (82.9%) conditions while RNLCs exhibited an increase in fluorescence intensity (depolarization) (91.5%) in dark conditions and a significant decrease in fluorescence (Hyperpolarization) (33.9%) when the cells were exposed to light and further hyperpolarized (14.6%) on continual light exposure

We analysed *PAX-6* expression in RNLCs by western blotting, ICC and flow cytometry. Around 83.2 ± 7.65% of RNLCs were PAX-6 positive (Fig. 3a), and we also found expression of *PAX-6* at protein levels (Fig. 3a). The ‘null’ expression of *PAX-6* on monocytes is depicted in supplementary Fig. 1, supplementary data. qPCR analysis indicated a significant upregulation in Recoverin (RCVRN) and IRBP mRNA levels and Rhodopsin protein level (Fig. 3b, c). It appears that the growth factors involved in differentiation of progenitor cells to photoreceptors (PR) for induction of RM towards retinal lineage might have induced the expression of PR markers in reconditioned monocytes (Supplementary Fig. 2).

Flow cytometric analysis of RNLCs stained with DiBAC_4_(3) exhibited an increase in fluorescent population in dark adapted RNLCs against a highly reduced fluorescent population in light exposed RNLCs at both time points (15 min and 30 min). DiBAC_4_(3) dye is a bis-oxonol dye that shows increased fluorescence upon depolarization and vice versa. However, RM cells exhibited no significant change between dark-adapted and light-exposed cells treated with the dye as compared to only dye treated control (Fig. 3d).

### RNLCs secrete neuroprotective signalling molecules in vitro

We analysed the secretion of neuroprotective paracrine signalling molecules and neurotransmitters including l-Glutamate, acetylcholine and nitric oxide (NO). These molecules are responsible for neuronal circuitry establishment in retina as well as cell-cell interactions. The expression levels of l-glutamate and Ach upregulated significantly at day 8 of RNLC stage as compared to RM at different time points (day 3 and day 6 of RM). Y-79 cell line was used as a positive control (Sup Fig. 4A, B). NO concentration also elevated at both day 4 and day 8 of RNLC differentiation culture (Sup Fig. 4C).

### RNLCs integrate into INL and GCL of immune compromised rd1 retina

**Fig. 4:**
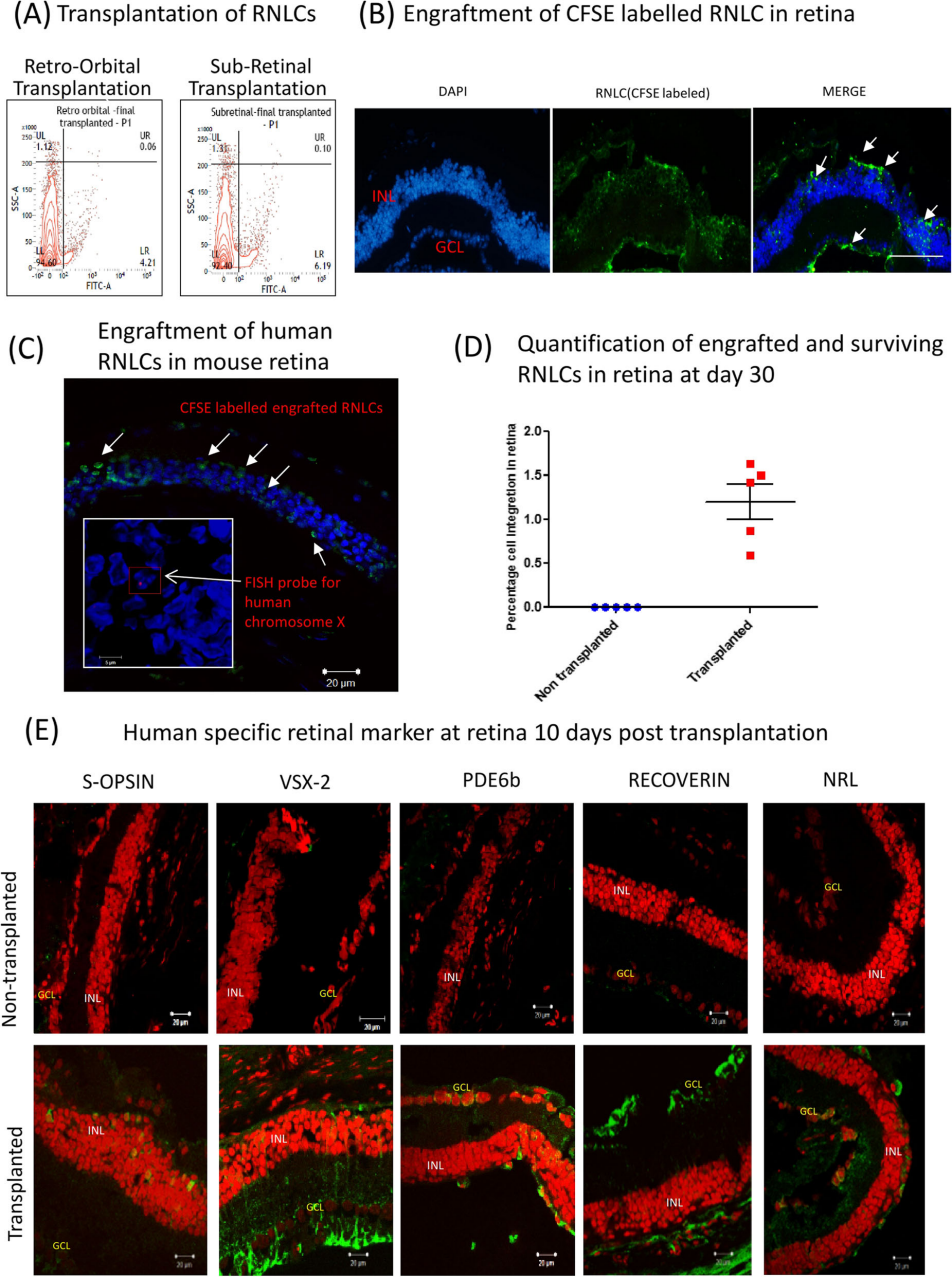
RNLC integration in immune compromised rd1mouse retina and further characterization. **a** The transplantation of RNLCs into the degenerated retina was optimized via subretinal injection as it offered higher transplantation efficiency (6.19% RNLCs in retina) than retro-orbital (4.21% RNLCs in retina as seen by flow cytometric analysis of CFSE-labelled human RNLCs cells gated in side scatter plot vs FITC. **b** The CFSE-labelled RNLCs could be visualized engrafted in the inner nuclear layer in rd1 retina stained with DAPI which further (**c**) labelled positive for human specific chromosome X using FISH probe. **d** qPCR was performed using GAPDH TaqMan probe to quantify the engrafted RNLCs. Around 1.2% of human cells were found to survive in the recipient mouse retina after 4 weeks of transplantation (*n* = 5). **e** IHC was performed for human-specific retinal markers in the retina of 10 days post transplanted animals. S-OPSIN-, PDE6b-, RCVRN- and NRL-positive cells were found in upper strata of INL while VSX-2-positive cells spanned across INL to GCL. Nuclei: PI (red), respective antibodies: Alexa fluor 488 (green)

CFSE-labelled RNLCs (1 × 10^6^) were initially transplanted retro-orbitally or subretinally in 4–6-week-old NOD.SCID-rd1 mice (*n* = 2) to optimize the route of injection for better engraftment. Post transplantation animals were checked for signs of ocular inflammation, and antibiotic eye drops were applied. The transplanted animals were euthanized after 3 days of injection. The retina was isolated and digested using Papain solution (10 μg/ml) to obtain single cell suspension. We found that approximately 6.19% RNLCs engrafted in retina upon subretinal and 4.21% engrafted upon retro-orbital injection (Fig. 4a). IHC studies also indicated that the CFSE-labelled RNLCs engrafted in the outer layers of INL after 48 h of transplantation in immune-deficient rd1 retina (Fig. 4b). The retina of transplanted mice also labelled positive for human-specific chromosome X suggesting that RNLCs derived from human monocytes could integrate and survive into degenerated rodent retina (Fig. 4c) as seen by FISH analysis. However, qPCR analysis using TaqMan probe specific for human GAPDH suggested that only 1.25 ± 0.447% (*n* = 5) of transplanted cells survived. Ten days after injection (Fig. 4d), the transplanted retina were analysed and it was found that the cells had integrated into mostly in INL and occasionally in GCL, and expressed retinal markers (*S-OPSIN*, *VSX2*, *GFAP*, *RCVRN* and *PDE6b*) (Fig. 4e). As a negative control, monocytes were transplanted and processed in an exactly similar manner and sections were stained for *S-OPSIN*, *RCVRN and PDE6b*. These sections were ‘clear’ and no stain was observed. Images are shown in supplementary Fig. 3, supplementary data file.

### cGMP levels decrease in both the eye and serum of transplanted animals

**Fig. 5:**
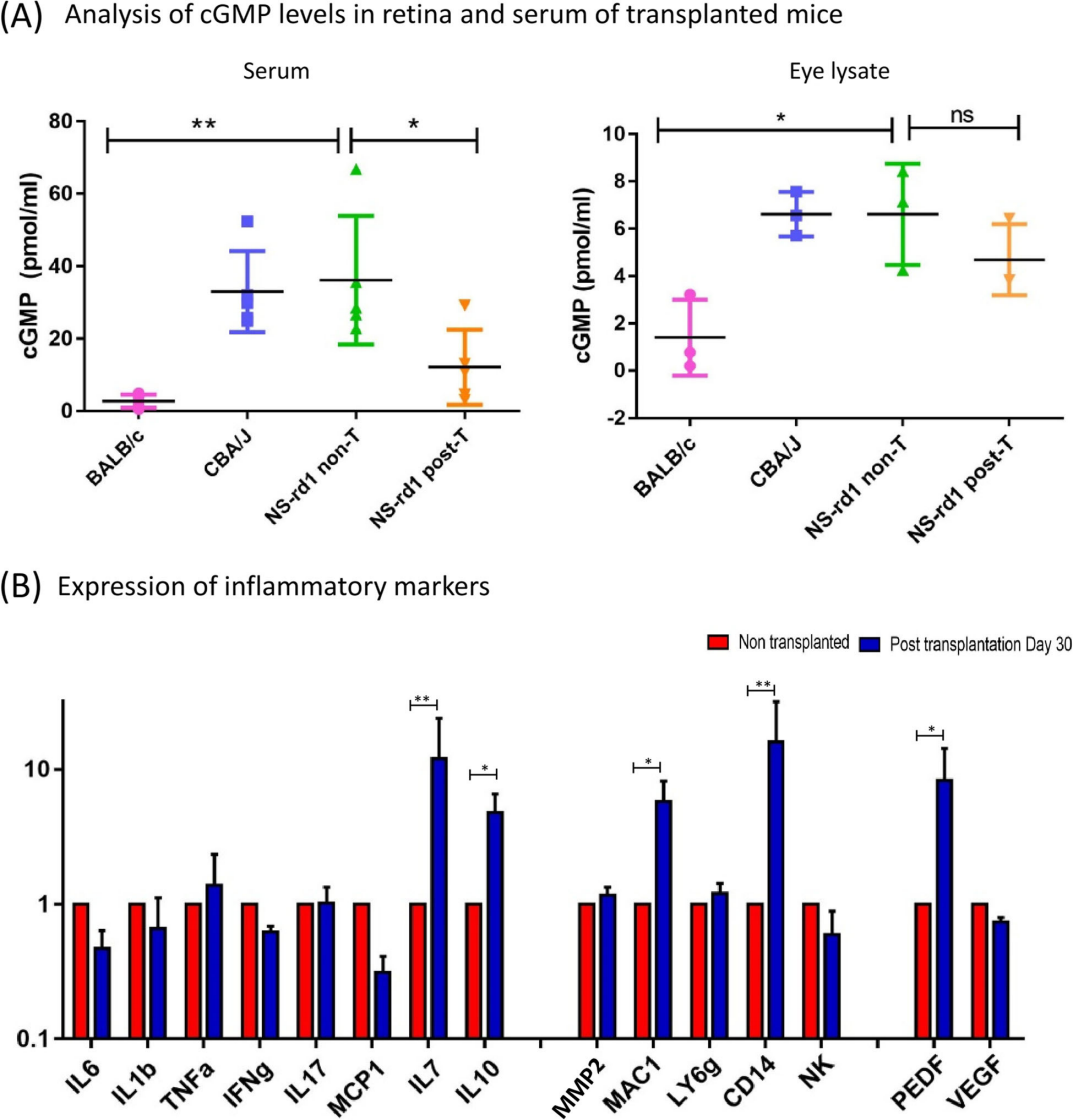
Analysis of transplanted animals for cGMP levels and inflammatory markers. **a** The serum level of cGMP was significantly reduced in post transplanted animals eye lysate samples also exhibited a slight reduction in total cGMP content post transplantation. (wild type) BALB/c, (rd1 model) CBA/J, transplanted animals (NOD-SCID-rd1). **b** qPCR analysis in transplanted animals suggested that pro-inflammatory cytokines such as TNF-a while anti-inflammatory cytokines such as IL-7 and IL-10 were exacerbated. The expression of MAC-1- and CD14-positive cells were upregulated while NK, (T cells) CCL2 and (Neutrophils) Ly6G had no noticeable difference. PEDF, a neuroprotective growth factor, was upregulated while VEGF showed no change (*n* = 4)

Since Pde6b mutated rd1 animals lack in the function of c-GMP hydrolysis during photo transduction cascade, we checked the cGMP levels in mouse serum and eye lysate samples in non-transplanted and transplanted animals. We found a decreased serum concentration of c-GMP in transplanted animals after 10 days as compared to non-transplanted animals (Fig. 5a). BALB/c wild type mice served as positive control. The c-GMP level in eye lysate also reduced in transplanted animals. This might be due to intracellular calcium level changes after transplantation, which alters the saturated cGMP channels by reopening them to function under light adaptations [22]. cGMP levels could also have been modulated by the transplanted RNLCs owing to their monocytic origin. The monocytes release antioxidants which can reduce hypoxia and lower cGMP levels as reported earlier [23].

### Macrophage and monocyte expression are upregulated in transplanted retina

Immune cells like macrophages, NK cells and neutrophils infiltrate the retina during retinal degeneration and inflammatory cytokines flare up causing inflammation [24–26]. Therefore, we studied the changes in immune cell population and inflammatory cytokines in the transplanted retina after 30 days to analyse the inflammatory responses in the retina after transplantation. We found a considerable increase in the levels of anti-inflammatory cytokine IL10 and IL7 and PEDF growth factor in the retina after transplantation; however, we also observed an elevation in the level of macrophage-related markers (MAC1 and CD14). Upregulation in macrophage markers indicates an immune activation response to RNLC transplantation and may play a major role in elimination of transplanted cells over time. We did not find any changes in T cell marker (CCL2), neutrophils (ly6G) and NK cell marker levels. (Fig. 5b).

### Behavioural studies post transplantation

**Fig. 6:**
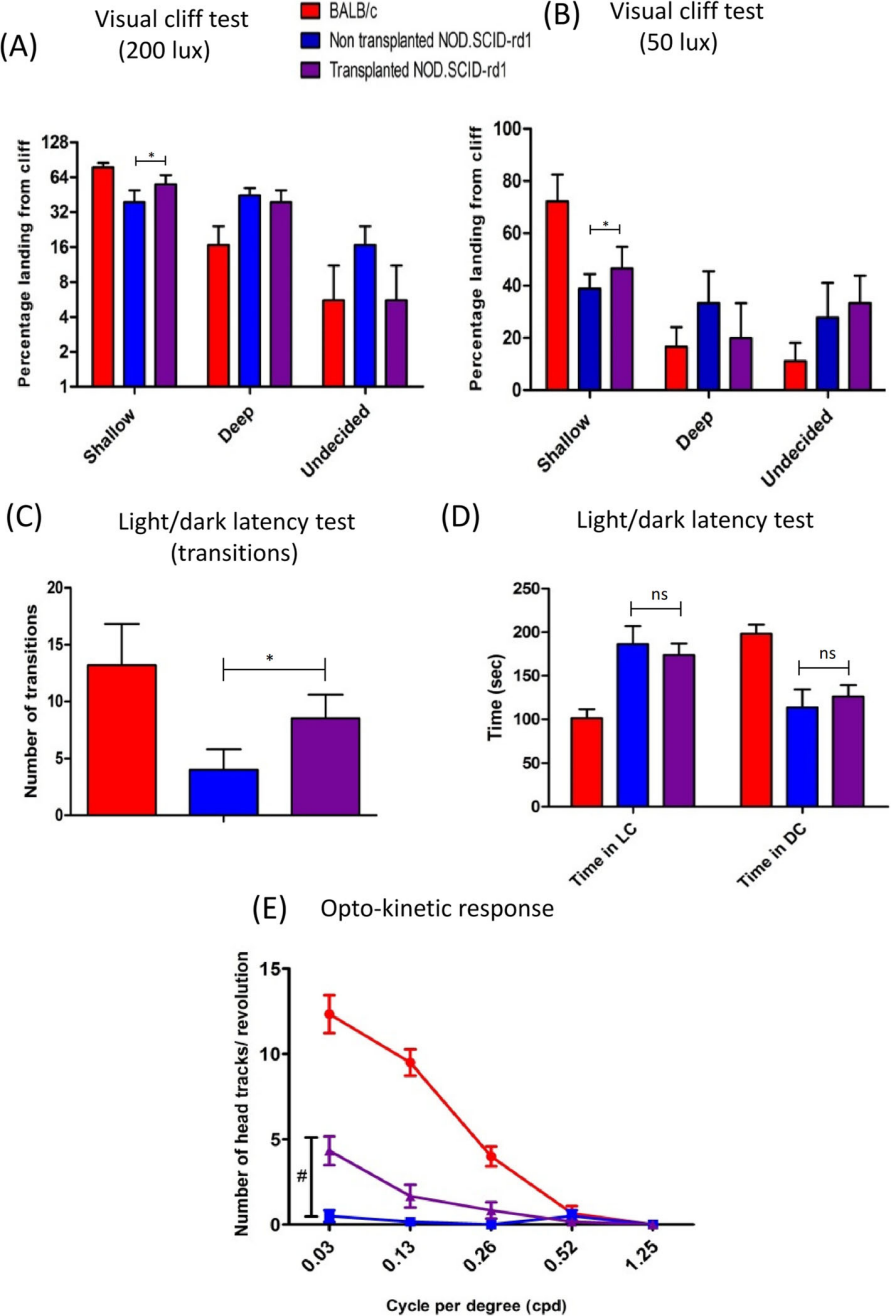
Functional analysis for vision rescue in host after transplantation of RNLCs in the retina. **a** The transplanted mice showed improved depth perception and stepped on the shallow side more number of times than deep side in both light (200 lx) and **b** dim (50 lx) conditions. **c** The light/dark latency test suggested that the transplanted animals spent comparable time in light chamber and that there was no noticeable change in their aversion to light as compared to non-transplanted animals. **d** The transplanted mice had an increased exploratory behaviour indicated by transitions between light chamber (LC) and dark chamber (DC) (*n* = 6). **e** The transplanted mice displayed significant increase in head tracks at 0.03 cycles per degree (cpd). It exhibited no significant change for the rest of the spatial frequencies (*n* = 6); *p < 0.05; #*p* < 0.001

The transplanted animals were studied for behavioural changes after transplantation, and traits like depth perception, optokinetic response, exploratory behaviour and light latency were examined. We observed that transplanted mice showed marked improved depth perception as evident by their capability to step on shallow side more number of times than deep side in both light (200 lx) and dark conditions (50 lx) (*p* < 0.05) (Fig. 6a, b). The transplanted animals also exhibited a better exploratory behaviour as they were able to locate the opening to the dark chamber easily and showed high number of transitions than non-transplanted mice (*P* < 0.05) (Fig. 6c). Nevertheless, their aversion to light still remained non-conclusive as they spent almost equal time in both the chambers and showed no significant improvement (Fig. 6d). We also observed a better response of transplanted animals to head tracking assessment in scotopic conditions (50 lx) at 0.03 cycles per degree (cpd) and 0.13 cpd (Fig. 6e). The changes between transplanted and non-transplanted animals at 0.03 cpd were significant (*p* < 0.0001).

## Discussion

In the present study, we used a two-step approach where human peripheral blood-derived monocytes were induced into RM and then differentiated with retinal growth factors towards neuronal cell type*.* Monocytes were reconditioned in culture conditions containing growth factors IL3 and MCSF and reducing agents like β-ME in a low serum environment for 6 days, initially demonstrated by Zhao et al. [27] and further studied extensively by Ruhnke et al. [19]. MCSF facilitates proliferation and differentiation of monocytes and IL3 is known to lead differentiation and self-renewal of pluripotent stem cells in presence of β-ME and low serum. Thus, this milieu induces pluripotency in monocytes. RM can be identified in culture as large adherent round cell population, sometimes forming colony structures, and are proliferative in nature.

The RM exhibited pluripotency markers like *CD34*, *CD117*, *OCT-4*, *SOX-2* and *NANOG* peaking at day 6 of de-differentiation. CD117 or c-KIT is the gene encoding the receptor tyrosine kinase (stem cell growth factor receptor) which is expressed on the surface of stem cells. CD34 is a progenitor cell marker while *OCT-4*, *SOX-2* and *NANOG* are transcription factors essential to maintaining the pluripotent embryonic stem cell phenotype [28]. Significant KI67 mRNA expression and incorporation of 5-ethynyl-2′-deoxyuridine (EDU) indicated pluripotency and proliferative capacity of RM. We also observed that the pluripotency in RM was transient and tends to decrease after day 6. The retrogression in the myeloid lineage observed in RM can be a function of reversible induction of cell cycle regulatory genes or epigenetic changes [29] such as methylation status of CSF-R responsible for de-differentiation [30, 31] and allow lineage-shift [32, 33]. However, the reconditioning of monocytes in this study does not involve manipulation of genes, rather is induced by extrinsic growth factor environment.

The induction of RM into RNLCs was achieved by treating them with a cocktail of growth factors involved in retinal cell differentiation [3]. Growth factors like b-FGF [3, 34] and EGF [35] are potent inducers of neuronal phenotype during cell cycle or immediately thereafter. Reports indicate that b-FGF especially induces the early response genes of retinal differentiation [34]. Retinoic acid and taurine play a crucial role in photoreceptor fate determination and differentiation [36–38]. Literature also suggests that IGF-1 and SCF signalling play a major role in the formation of three-dimensional ocular structures from human ESCs and its absence dramatically reduced the level of retinal progenitor genes [32, 38]. Mellough et al. have shown that B27 is a serum free supplement with various factors that assists differentiation of pluripotent cells to photoreceptors [38].

Upon induction for a period of 8 days, RNLCs exhibited morphological alteration showing elongated structure, neuron-like axonal spread out and cell-cell contacts. Additionally, we found a significant upregulation in neuro-signalling molecules like l-glutamate, Ach and NO that also act as paracrine factors in retina and facilitate cell interactions [39–41]. qPCR results showed that RNLCs also exhibited an upregulation in a few neuronal and retinal markers (IRBP and RHO mRNA). RNLCs expressed retinal markers PAX-6 and RHO at protein levels. Approximately 90% of RNLC population was PAX-6+ve suggesting that a large proportion of RM shifted towards ectodermal neuronal lineage which correlates to previous study by Kodama et al. [42] who demonstrated the capacity of monocyte-derived multipotential cells (MOMC) to differentiate into neuroectodermal lineages suggesting that circulating CD14+ monocytes are highly multipotential.

The RNLCs also demonstrated membrane potential changes during light and dark adaptations as measured by changes in DiBAC4(3) fluorescence intensity which was largely a relative comparison between RM and RNLC [43, 44]. It has been reported earlier that bis-oxonol dyes may sometimes show artefacts and lower temporal resolution in measurement of membrane potential [45] and that the demonstration of fluorescence intensity changes in DiBAC4(3) associated with membrane potential is largely relative rather than quantitative in our current study.

Transplantation of RNLCs in immune compromised NOD.SCID-rd1 mice via trans-corneal sub-retinal transplantation resulted in their engraftment owing to proximity between damaged layer and site of transplanted cells [2] and that it is more immune privileged than other retinal regions [46]. As previously shown in our lab and by other groups, the cell engraftment efficiency in an immune-deficient rd1 model is higher than an immune-competent rd1 model [20, 47, 48]. Therefore, we performed cell transplantation studies on immunodeficient rd1 mouse model to assess cell integration.

Around 1.2 ± 0.447% cells of total transplanted CFSE-labelled RNLCs survived in the retina after 4 weeks of transplantation. The engrafted RNLCs expressed human-specific retinal markers like RCVRN, NRL (early PR markers), VSX2 (Pan-bipolar cell marker), PDE6B (phototransduction marker) and S-OPSIN (cone PR marker) dispersed throughout the INL and GCL. The monocyte transplanted retina however did not show any retinal marker expression. Therefore, we speculate that the in vivo ocular microenvironment helps the already induced RNLCs to integrate and further acquire retinal cell-like characteristics after transplantation.

The transplanted animals displayed lower levels of cGMP in both eye lysate as well in serum after 10 days of transplantation. This might be due to intracellular calcium changes altering the saturated cGMP channels. Alternatively, it is known that monocytes release antioxidants which can reduce hypoxia and lower cGMP levels as reported earlier [23]. Therefore, the monocytic origin of induced RNLCs may have caused the lowering of cGMP levels. Immune cells including *MAC-1*- and *CD14*-positive cell mRNA (macrophages and monocytes respectively) were increased, suggesting that these inflammatory cells were activated in retina following transplantation. PEDF and IL-10 were also upregulated in transplanted animals indicating the highly active neuroprotection activity in retina after transplantation. The rd1 model used for transplantation was devoid of B and T cells and had a reduced NK cell activity (NOD.SCID-rd1). This explains the fact that there was no NK cell activity.

The behavioural changes in transplanted mice indicated that they had improved depth perception in both light and dim conditions as indicated by their ability to step on shallow side of the cliff. The transplanted mice also had an improved exploratory behaviour than non-transplanted animals while their behaviour study showing aversion to light was not conclusive. We also observed significantly improved optokinetic response in the transplanted mice at 0.03 cpd. Even though the behavioural changes in the transplanted animals are promising, we speculate that the percentage of transplanted cells (1.25%) that engrafted is insufficient for cell-based therapeutic effect and for structural rescue. Previous studies on cell therapy have encountered similar situations where the engraftment of cells is low, but they observed significant therapeutic efficacy, which was attributed to paracrine activity of cells and their capability to secrete neuroprotective cytokines and growth factors [49, 50].

In the present study, we have not investigated a direct evidence for the paracrine mechanism upon cell transplantation. However, several cases in our study indirectly point towards elicitation of paracrine effect in the retina upon transplantation of RNLCs. We observe an elevation of IL-10 (an anti-inflammatory cytokine) in retina upon transplantation which suggests immune-protective effect of RNLCs. We also found upregulated PEDF growth factor (involved in anti-angiogenesis and neuroprotective functions for photoreceptor cells and retina) in transplanted retina which points towards involvement of paracrine-based protective role of RNLCs in retina.

We also find that RNLCs have the potential to secrete neurotransmitters like nitric oxide, acetylcholine and l-glutamate in vitro, which also have a broad range of paracrine signalling function in neurons besides synaptic functions [39–41] (Sup Fig. 4).

We speculate that RNLCs act in paracrine fashion and secrete neuronal growth factors which help in sustenance and survival of retinal cells and function in stress conditions. Several groups have previously shown that dedifferentiated monocytes with pluripotent and plastic nature can secrete EGF, TGF-β and Activin in vitro [51]. We are also currently studying the underlying mechanisms for therapeutic effect of RNLC transplantation.

Our current findings suggest that monocytes can be reconditioned to elicit pluripotency and proliferation and induced towards neuronal lineage extrinsically. The induced RNLCs have the ability to integrate into retina of immune-deficient rd1 mice. RNLCs can also utilize the retinal microenvironment to supplement their further differentiation and survival into retinal layers and express retinal markers. However, this preliminary study is limited by a small group of animals, and further mechanistic and quantitative functional assessment in a larger number of animals need to be performed to confirm if induced RNLCs could potentially be used for long-term therapeutics in retinal degenerative disorders.

## Conclusion

Monocytes were cultured in a defined medium to create the right environment to elicit their physiological pluripotent capacities. The pluripotent cells of monocyte origin (RM) are adherent, colony forming cell population and exhibit transient pluripotency and proliferation. We induced RM towards a neuronal lineage by culturing them with a cocktail of growth factors involved in retinal cell differentiation. The neuronal induction causes morphological, physiological and molecular alterations in RM, and these cells have reduced expression of monocytic and myeloid markers and upregulated ectodermal and retinal neuron markers. Transplantation of RNLCs in immune compromised NOD.SCID-rd1 mice via trans-corneal subretinal transplantation resulted in their engraftment in INL and GCL. It also led to macrophage/monocyte upregulation. The transplanted mice demonstrated slightly improved depth perception in both light and dim conditions and a better optokinetic response. In this study, we successfully demonstrated that abundantly accessible peripheral blood-derived monocytes, which are highly abundant and can be obtained by minimal invasive procedures, can be inducted towards neuronal lineage. However, we need further studies to explore the functional activity and in vivo differentiation capacity of the induced RNLCs. It would establish monocyte-derived induced RNLCs as a potential candidate for future cell therapy studies in ocular diseases, and their utilization for disease modelling and drug screening purposes.

## Supplementary information

**Additional file 1.** Detailed methods and additional data are given in the Supplementary data file.

## Data Availability

All data generated or analysed during this study are included in this published article and its supplementary data file.

## Abbreviations

EDU: 5-Ethynyl-2′-deoxyuridine
b-FGF: Basis fibroblast growth factor
CFSE: Carboxyflourescein succinimidyl ester
cpd: Cycles per degree
DP: Depolarization
ESC: Embryonic stem cells
EGF: Epidermal growth factor
FBS: Fetal bovine serum
GCL: Ganglion cell layer
HP: Hyperpolarization
iPSC: Induced pluripotent stem cells
INL: Inner nuclear layer
IGF1: Insulin growth factor1
IL: Interleukin
MCSF: Macrophage colony-stimulating factor
NK: Natural killer cells
NRL: Neural retina-specific leucine zipper
NEAA: Non-essential amino acid
PFA: Paraformaldehyde
PBMC: Peripheral blood mononuclear cells
PR: Photoreceptors
PEDF: Pigment epithelium-derived factor
PI: Propidium iodide
RM: Reconditioned monocytes
RCVRN: Recoverin
RNLC: Retinal neuron-like cells
RP: Retinitis pigmentosa
RA: Retinoic acid
RHO: Rhodopsin
RT: Room temperature
SCF: Stem cell factor
PDE6B: β-subunits of phosphodiesterase 6
