# Supplementary material for: Peripheral blood-derived monocytes show neuronal properties and integration in immune-deficient rd1 mouse model upon phenotypic differentiation and induction with retinal growth factors
Source: Stem Cell Res Ther. 2020 Sep 23;11:412. doi: 10.1186/s13287-020-01925-y (PMC7510317; doi:10.1186/s13287-020-01925-y)
Supplement: Supplementary file 1 — Additional file 1. Detailed methods and additional data are given in the Supplementary data file. [file 13287_2020_1925_MOESM1_ESM.docx]

**Supplementary data file**

Peripheral Blood Derived Monocytes show neuronal properties and integration in immune-deficient rd1 mouse model upon phenotypic differentiation and induction with retinal growth factors

**List of contents**

*Item Page*

Materials and Methods 2

(A) Scanning Electron Microscopy 2

(B) Human specific GAPDH TaqMan probe 3

(C) Fluorescence In Situ Hybridization (FISH) 4

(D) Behavioral Analysis 5

(E) Analysis of neuro-protective/ paracrine signaling molecules in vitro 7

Table 1: Primary antibodies 8

Table 2: List of human myeloid / monocytic lineage genes 9

Table 3: List of human ectodermal lineage genes 10

Table 4: List of human retina specific genes 10

Table 5: List of human pluripotency and proliferation genes 11

Table 6: List of human monocyte and housekeeping gene 11

Table 7: List of mouse immune cells and inflammatory genes 12

Table 8: List of manufacturers for products used 13

Sup Fig 1: Expression of PAX-6 on monocytes 16

Sup Fig 2: Expression of neuronal markers in RNLCs 17

Sup Fig 3: Retinal marker analysis in monocyte transplanted mice 18

Sup Fig 4: Analysis of paracrine signaling molecules in RNLC 19

**Materials and Methods**

1. **Scanning Electron Microscopy**

SEM was performed to analyze the morphological and structural changes over time during two step differentiation from monocyte to RNLCs. The cells (monocytes, DM and RNLCs) were cultured on human plasma fibronectin (5ug/cm^2^) coated coverslips and media was washed off the coverslips with PBS. The cells were fixed in SEM fixative for 4 hours at RT followed by three washes with sodium cacodylate buffer. The cells were then treated with 1% osmium tetrachloride for 1 hour followed by a series of alcohol dehydrations.

| Sr No | Alcohol (%) | Time |
| --- | --- | --- |
| 1 | 25% ethanol | 5 minutes |
| 2 | 50% ethanol | 7 minutes |
| 3 | 70% ethanol | 10 minutes |
| 4 | 95% ethanol | 20 minutes |
| 5 | 100% ethanol | 30 minutes (X 3) |

The cells were treated with hexamethyldisilazane (HMDS) (Sigma, USA) for 15 minutes after dehydration and they were attached to stubs using double sided carbon tape. The samples were then sputter coated with gold-palladium alloy in a mini sputter coater (Electron Microscopy Sciences, USA). The coated samples were finally viewed under scanning electron microscope and representative images were captured. The composition of the buffers used are given below:

1. ***Cacodylate Buffer:***

0.4M solution of Sodium Cacodylate

Na(CH_3_)_2_AsO_2_.3H_2_0 (21.4 g)

Add distilled water to make 250 ml.

0.2M Cacodylate Buffer, pH 7.2

0.4M Sodium Cacodylate (50 ml)

0.2M HCl (8 ml)

Add distilled water to a final volume of 100 ml.

The pH of the buffer is adjusted, if necessary, to the required value with HCl.

1. ***4% Glutaraldehyde:***

0.2M buffer (50 ml)

25% glutaraldehyde (16 ml)

Add distilled water to a final volume of 100 ml

1. ***SEM Fixative:***

0.2M buffer (50 ml)

37% formaldehyde (11 ml)

25% glutaraldehyde (4 ml)

Add distilled water to a final volume of 100 ml

This fixative contains 4% formaldehyde and 1% glutaraldehyde in 0.1M buffer

1. ***1% Aqueous Osmium tetroxide:***

Mix equal parts of 2% aqueous stock osmium tetroxide and 0.2M phosphate buffer to obtain 1% osmium tetroxide in 0.1M phosphate buffer.

1. **qPCR using human specific GAPDH TaqMan probe for estimation of cell integration upon transplantation in retina**

qPCR was performed using a Taqman probe which was specific to human GAPDH gene. We checked cross reactivity to mouse cDNA and did not find any cross reactivity. The probe was conjugated to a fluorescent dye (6-carboxyfluorescein or FAM) at 5’ end and a quencher (tetramethylrhodamine or TAMRA) on the 3’ end. When the probe was bound to the specific template, the exonuclease activity of 5’ -DNA polymerase released the quencher thereby activating the fluorescence of the amplified product.

The following table enlists the reagents used for qPCR using Taqman probe.

| **Serial No.** | **Reagents** | **Volume** |
| --- | --- | --- |
| 1. | 2 X qPCR Master Mix (Thermo Scientific, USA) | 12.5μl |
| 2. | Template cDNA | 2.5μl |
| 3. | Solaris Primer/Probe Set (20X) (Thermo Scientific, USA) | 1.25μl |
| 5. | Molecular Biology Grade water | 8.75μl |

PCR conditions used:

| **Serial No.** | **Operation** | **Temperature** | **Time** | **Cycle** |
| --- | --- | --- | --- | --- |
| 1. | Enzyme Activation | 95^o^C | 15 minutes | 1 cycle |
| 2. | Denaturation | 95^o^C | 15 seconds | 40 cycle |
| 3. | Annealing and Elongation | 60^o^C | 60 seconds |  |

**C) Fluorescence In Situ Hybridization (FISH)**

The animals were euthanized and eyes were enucleated. The eyes were then washed and fixed in freshly prepared 4% PFA for 4-24 hours at 4°C. The tissues were incubated in an increasing sucrose concentration from 10 % to 30% for 1 hour each. The tissue samples were embedded in OCT, frozen at cryobar temperature and sectioned to obtain 5 um sections on L-polylysine coated slides. The slides were again transferred into a coplin jar with prewarmed (80°C) sodium citrate buffer (10mM) for 5-25 minutes then cooled to room temperature. The slides were incubated in 2X SSC buffer for 5 minutes. The slides were then transferred to 50% formamide in 2X SSC and incubated for 1 hour. Hybridization chamber was prepared from glass coverslip (8mm X 8mm) by cutting it into equal sized pieces and glued them to a coverslip of same size parallel to each other on the opposite sides using nail polish. The DNA probe was added to the tissue section slowly to fill the chamber and the chamber was sealed using rubber cement. The hybridized samples were placed on a hot-block (or water bath) with temperature 45°C for 1–3 hours in order to allow permeation of the section with the probe. The slides were thereafter placed on a hot-block at 80°C for 5 minutes for the probe and section denaturation of DNA. Then the slides were further hybridized at 37°C for 2 to 3 days in a temperature-controlled oven. The slides were washed in 2X SSC three times for 10 minutes each to remove excess hybridization mixture properly. Stringent washes were performed in 0.1X SSC at 60°C, 2×5 minutes to remove nonspecific hybrids. Then the slides were equilibrated in 2X SSC for 2 minutes followed by secondary antibody (Streptavidin Alexa Fluor 594) staining for 30 minutes, 3 washes in 2X SSC for 10 minutes each and counterstaining with DAPI (50ug/ml) for 5 minutes. The slides were mounted with coverslip in vectashield or 50% glycerol and sealed from all sides using nail polish. The sections were visualized and imaged in confocal microscope at 63X magnification.

**D) Behavioral Analysis**

1. **Optokinetic response (OKR)**

The Optokinetic drum was made of a rotating drum with stripes of varying grating frequencies (0.03, 0.13, 0.26, 0.52 and 1.25 cpd) which contained a wooden platform at the center, 15 cm above the base and 10 cm from to the periphery of the drum (diameter: 63 cm; height: 35 cm). The animals were preconditioned to the non-rotating apparatus by leaving them on the platform for 5-10 minutes before the actual experiment. Ambient room light (illuminance = 400 lux) was maintained in the center of the cylinder and measured with a digital luxmeter (MASTECH®). The optokinetic drum was set to rotation speed of 2 rpm and a spatial frequency of 0.1 cyc/deg. The animals were placed on the platform and observed until they become stable. Thereafter the drum was rotated clockwise and anticlockwise, 1 minute each for every stripe used with a time interval of 30 seconds between two rotations. Head-tracking reflex was judged by the observer (horizontal head movement at the same rate and the same direction as the drum for at least 15°). If the spatial frequency of the black and white stripes was increased, a threshold was reached beyond which no tracking movements of the head were detected. The visual spatial resolution ([visual acuity](http://topics.sciencedirect.com/topics/page/Visual_acuity)) of the animal was estimated to be greater than or equal to this threshold but below the next spatial frequency tested.

1. **Visual cliff test**

A wooden box of dimensions measuring 62 x 62 x62 cm (l x b x h) was used to construct a visual cliff apparatus. The four edges emerged at least 19 cm above the top and a wooden platform was placed exactly in the middle of the apparatus. The platform was 3.75 cm in height, 60 cm in length and 2.5 cm in width. The box was separated in two sections using the platform, one of which was covered with checker paper placed on the top surface (made of glass) of the box which was considered as shallow or safe zone whereas the other side had the same checker board paper placed on the bottom surface of the box and appeared to be the deep zone or cliff zone. To analyze the vision and depth perception of mice, the mouse was placed on the platform and observed for mouse to decide to step towards either safe zone or cliff zone in 5 minutes. Individual mouse of each strain was given 5 trials after preconditioning to the apparatus. To reduce the effect of learning and memory, the apparatus was turned 180 degrees after two trials so that the “safe zone” and the “cliff zone” were at different sides for the next three trials. The glass surface was wiped thoroughly each time to prevent mice from finding odor clues. The number of times the mouse stepped down to shallow zone and cliff zone was counted and percentage of stepping towards each zone (shallow /deep) was calculated.


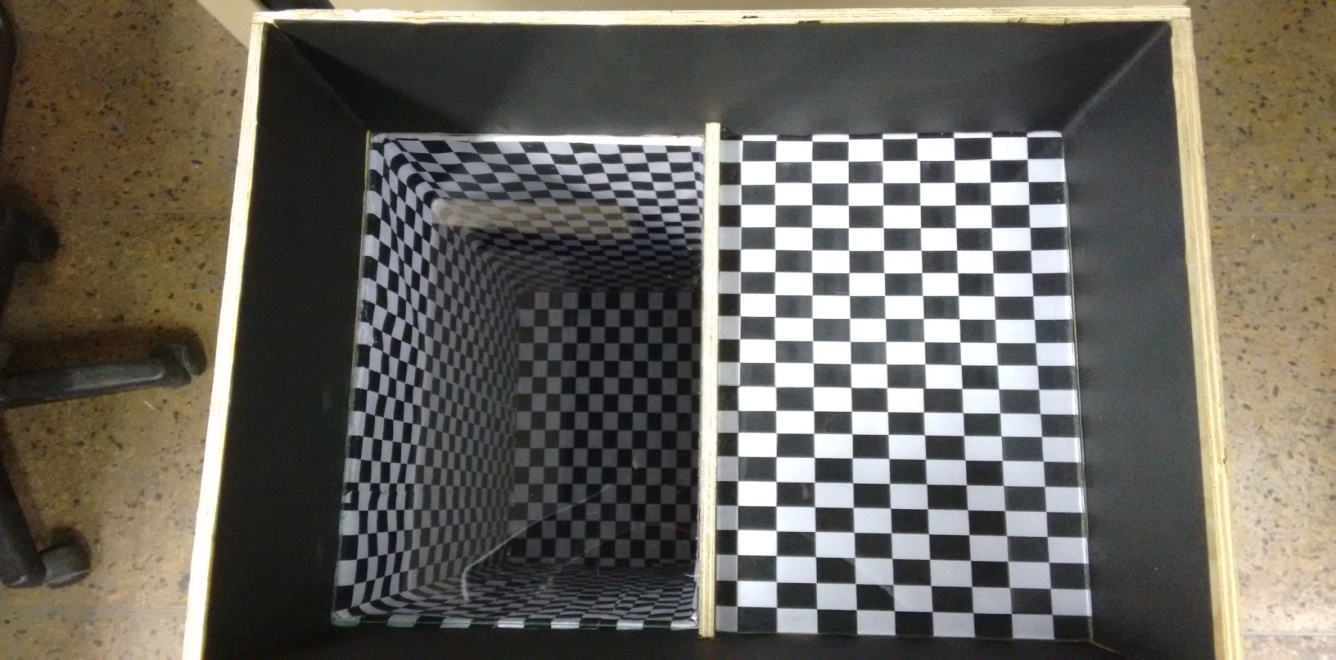


**Deep**

**Shallow**

**Cliff**

Representative image of Visual cliff apparatus

1. **Light / dark latency test**

A box sized 21 X 42 X 25cm was equally divided in two parts by a partition wall with a 5 cm connecting opening. This apparatus was called Light/Dark apparatus as one of the chambers was brightly illuminated (approx. 400 lux) with white colored background and the other was kept completely dark with black background. The animal was introduced in the light chamber (LC) and observed for five minutes. The total time spent by the animal in each chamber was recorded along with the number of transitions from LC to DC and vice versa. The initial time taken by the animal to enter the DC from LC where it was placed was also noted down.

**E) Analysis of neuro-protective/ paracrine signaling / neurotransmitter molecules in vitro**

1. **L - Glutamate estimation**

Standards from 0 μM to 20 μM were prepared by diluting appropriate amount of 200 mM L - glutamic acid stock solution in the provided 1x reaction buffer. H_2_O_2_ (10 μM) diluted in 1x reaction buffer was used as a positive control while 1x reaction buffer alone was used as blank. A working solution of 100 μM Amplex® Red reagent containing 0.25 U/ml HRP, 0.08 U/ml L - glutamate oxidase, 0.5 U/ml L - glutamate - pyruvate transaminase and 200 μM L - alanine was prepared just prior to the experiment. 50 μL of the standards, culture supernatant samples and controls was dispensed into separate wells of a 96 well microplate followed by addition of 50 μL of Amplex® Red reagent solution. The plate was incubated for 30 minutes at 37oC in dark. Thereafter, the fluorescence was measured in a fluorimeter using excitation wavelength of 530 - 560 nm, while the emission was detected at 590 nm. The standard curve was plotted using the fluorescence readings of the standards after background correction and the value for each sample was calculated.

1. **Acetylcholine estimation**

Acetylcholine standards from 0 μM to 100 μM were prepared by diluting appropriate amount of 100 mM acetylcholine stock solution using the provided 1x reaction buffer. The 1x reaction buffer alone was used as blank. The positive control was prepared by diluting 20 mM H2O2 in 1x reaction buffer to yield 10 μM solution. The culture supernatant samples were appropriately diluted to fall in the range of standards. 100 μL of samples, standards and controls were dispensed into separate wells of a 96 well microplate. 100 μL of a freshly prepared solution of 400 μM Amplex® Red reagent containing 2 U/ml HRP, 0.2 U/ml choline oxidase and 1 U/ml acetylcholinesterase was added in each well and the plate was incubated for 30 minutes at RT in dark. After incubation, the fluorescence was measured in a fluorescence microplate reader at 590 nm. The background fluorescence was corrected and standard curve was plotted. The concentration of acetylcholine in each sample was calculated accordingly.

1. **Nitric oxide / Nitric oxide synthase (NO / NOS) estimation**

Culture supernatant was collected at different time points and diluted appropriately in assay diluent (10% FBS in PBS). Standards ranging from 0 μM to 100 μM were prepared by serially diluting Sodium nitrite (NaNO2) in assay diluent. Griess reagent was prepared by adding solution A [N-(1-Naphthyl) ethylenediaminedihydrochloride or NED] and solution B (p-aminobenzenesulfonamide or Sulfanilinamide) in equal proportions. 50 μL of standard or sample was added per well of a 96 well microplate, followed by addition of 50 μL Griess reagent in each well. The absorbance reading was taken immediately at 540 nm using a microplate reader. The standard curve was plotted and concentration of NO/NOS was calculated in each sample.

**Table 1: Primary and secondary antibodies for Flow cytometry, Western blotting, Immunohistochemistry and Immunocytochemistry studies.**

| **Sr. No** | **Antibody** | **Fluorochrome** | **Working Concentration** |
| --- | --- | --- | --- |
|  | *CD14* | FITC/ PECy7 | 1:400 (FACS) |
|  | *RHODOPSIN* | Purified | 1:200 (IHC), 1:500 (WB) |
|  | *PAX-6* | Purified | 1:200 (IHC), 1:500 (WB) |
|  | *CD117* | PECy7 | 1:200 (FACS) |
|  | *RECOVERIN* | Purified | 1:200 (IHC) |
|  | *NRL* | Purified | 1:200 (IHC) |
|  | *S-OPSIN* | Purified | 1:100 (IHC) |
|  | *VSX-2* | Purified | 1:200 (IHC) |
|  | *PDE6b* | Purified | 1:200 (IHC) |
|  | *β-ACTIN* | Purified | 1:500 (WB) |
|  | *Alexafluor 488 goat-anti mouse IgG* | *Alexafluor 488* | 1:500 |
|  | *Alexafluor 594 goat-anti rabbit IgG* | *Alexafluor 594* | 1:500 |
|  | *Alexafluor 594 goat-anti-rat IgG* | *Alexafluor 594* | 1:500 |

**Table 2: List of human myeloid / monocytic lineage genes**

| **Sr. No.** | **Gene name** | **Forward Primer (5’-3’)** | **Reverse Primer (5’-3’)** | **Amplicon Size (bp)** |
| --- | --- | --- | --- | --- |
| 1 | *CD14* | GAGTCAACAGGGCATTCA | ACCGTAACAGGAAGGATTC | 100 |
| 2 | *CD53* | TCTACCTGCTGATCCACAAC | GCCCACGATGACAAACAC | 86 |
| 3 | *CD163* | ACTGGACCGATATGGCTCAA | GCGTCTGGCAGGACAATC | 75 |
| 4 | *CX3CR1* | GAGGCTGGTTCTTACGATGG | GGAGAGTTGGGTTACGGAAG | 77 |
| 5 | *CXCR4* | ATCTGTGACCGCTTCTACC | GATGACAATACCAGGCAGGA | 93 |
| 6 | *CXCR6* | ACAACCAGCAAGCCAAGA | CAAGGAAACCAGCAGGGATA | 80 |
| 7 | *CD45* | GCACAGGAACCTATATCGGAAT | CTTGAACCATCAGGCATCTCT | 117 |
| 8 | *CD115* | GAGTTGACGACAGGGAGTA | ATGACCGAAGGCAGAGTT | 100 |
| 9 | *CD56* | TGCTGAGTATGAGGTCTACG | CCGAGGTCCTGAACACAA | 80 |
| 10 | *CD192* | GCTGCTCATCATGGTCATC | ACTCTCACTGCCCTATGC | 90 |
| 11 | *CD11b* | TATGACCTGGCTCTGGACTC | TCTGCGTGTGCTGTTCTT | 75 |

**Table 3: List of human ectodermal lineage genes**

| **Sr. No.** | **Gene name** | **Forward Primer (5’-3’)** | **Reverse Primer (5’-3’)** | **Amplicon Size (bp)** |
| --- | --- | --- | --- | --- |
| 1 | *PAX-6* | AGTGTCTACCAACCAATTCCA | GCTGTAGGTGTTTGTGAGG | 102 |
| 2 | *CHRD* | TGGAGAGATGAGCTGTATCAC | ACAGTGGCAGTGAACAGT | 80 |
| 3 | *bFGF* | TGCATGAACAAGAAGGGGAAGC | TCCAGCACAATCTCCGTGAAG | 80 |
| 4 | *FOXJ3* | CTCAGCACTCCAGGAACAAC | AGGCTTGGGAAGGCATCAAT | 76 |
| 5 | *TUBB* | GATCAGCGTCTACTACAAC | TGAAGAGATGTCCAAAGG | 119 |
| 6 | *SOX-2* | ACAACTCGGAGATCAGCA | TTAGCCTCGTCGATGAAC | 85 |
| 7 | *PAX-2* | AGGCATCAGAGCACATCA | TTGGTGGATGCAGATAGACT | 103 |
| 8 | *OTX-2* | CCAGACATCTTCATGCGAGAG | TGTTGTTGGCGGCACTTAG | 104 |

**Table 4: List of human retina specific genes**

| **Sr. No.** | **Gene name** | **Forward Primer (5’-3’)** | **Reverse Primer (5’-3’)** | **Amplicon Size (bp)** |
| --- | --- | --- | --- | --- |
| 1 | GFAP | CCACCTCAAGAGGAACATC | CCTGCCTCACATCACATC | 101 |
| 2 | IRBP | CTTGTGCTCCTACTTCTTTGA | CCATAGCGTTCACCTACA | 120 |
| 3 | PAX-6 | GTGAATGGCCGGAGTTATG | TGAGTCCTGTTGAAGTGGT | 100 |
| 4 | SYNPT | GCTGTGTTCGCCTTCATG | TCTCCTTGATAATGTTCTCTGGG | 100 |
| 5 | TUBB | GATCAGCGTCTACTACAAC | TGAAGAGATGTCCAAAGG | 119 |
| 6 | RPE-65 | CAATGGGTTTCTGATTGTGGA | CCAGTTCTCACGTAAATTGGCTA | 91 |
| 7 | NES | CTCCAAGACTTCCCTCAG | GAGCAAAGATCCAAGACG | 88 |
| 8 | RCVRN | CCAGAGCATCTACGCCAAGT | CACGTCGTAGAGGGAGAA | 187 |

**Table 5: List of human pluripotency and proliferation genes**

| **Sr. No.** | **Gene name** | **Forward Primer (5’-3’)** | **Reverse Primer (5’-3’)** | **Amplicon Size (bp)** |
| --- | --- | --- | --- | --- |
| 1 | CD34 | CCTCCCAAGTTTTAGGACAA | CAGCTGGTGATAAGGGTTAG | 362 |
| 2 | NANOG | AATAGCAATGGTGTGACGCAGAA | GGCATCCCTGGTGGTAGG | 76 |
| 3 | c-MYC | CTGCTTAGACGCTGGATT | CATAGTTCCTGTTGGTGAAG | 93 |
| 4 | KI67 | CGACCCTACAGAGTGCTCAACAAC | AACTGCGGTTGCTCCTTCACT | 113 |

**Table 6: List of human monocyte and housekeeping gene**

| **Sr. No.** | **Gene** | **Forward Primer (5’-3’)** | **Reverse Primer (5’-3’)** | **Amplicon Size (bp)** |
| --- | --- | --- | --- | --- |
| 1 | CD14 | AGGTTCGGAAGACTTATC | GAAATCTTCATCGTCCAG | 118 |
| 2 | GAPDH | CAACAGCCTCAAGATCATCAG | GAGTCCTTCCACGATACCAA | 100 |

**Table 7: List of mouse immune cells and inflammatory genes**

| **Sr. No.** | **Gene name** | **Forward Primer (5’-3’)** | **Reverse Primer (5’-3’)** | **Amplicon Size (bp)** |
| --- | --- | --- | --- | --- |
| 1 | Mac-1 | TTCACGGCTTCAGAGATG | CCATACGGTCACATTGTTG | 129 |
| 2 | Ly6g | CGTTGCTCTGGAGATAGAAGTTA | GTTGACAGCATTACCAGTGAT | 106 |
| 3 | Nk | GGTTCTGGACAAGATGAAGT | CGATGCCGATGTTGATGA | 101 |
| 4 | Mmp2 | GACCACAACCAACTACGATGA | GCTGCCACGAGGAATAGG | 78 |
| 5 | Mcp1 | CTACTCATTCACCAGCAAGAT | TCAGCACAGACCTCTCTC | 126 |
| 6 | Pedf | CTTACGATACGGCTTGGA | CTTGGATAGTCTTCAGTTCTC | 179 |
| 7 | Vegf | CGACAGAAGGAGAGCAGAAG | CTCAATCGGACGGCAGTAG | 80 |
| 8 | IL17 | CTCACACGAGGCACAAGT | GCAGCAACAGCATCAGAGA | 97 |
| 9 | IL10 | CTGGGTGAGGAAGCTGAAG | CCACTGCCTTGCTCTTAT | 85 |
| 10 | IL7 | ATCCTTGTTCTGCTGCCTGTC | TTCGGGCAATTACTATCAGTTCCT | 134 |
| 11 | IL1b | AAGGGCTGCTTCCAAACC | GATGTGCTGCTGCGAGAT | 75 |
| 12 | IL6 | CGCTATGAAGTTCCTCTC | TCTGTGAAGTCTCCTCTC | 114 |
| 13 | Tnf-A | CACCACCATCAAGGACTCA | GGCAACCTGACCACTCTC | 109 |
| 14 | Ifn-g | CTGAGACAATGAACGCTACAC | TCTTCCACATCTATGCCACTT | 145 |

**Table 8: List of products used and their manufacturer information**

| Sr. No | Product | Manufacturer |
| --- | --- | --- |
|  | PBS | Himedia, India |
|  | IMDM | Himedia, India |
|  | FBS | Gibco, USA |
|  | TRYPSIN | Himedia, India |
|  | TRYPAN BLUE | Himedia, India |
|  | 12 WELL CULTURE PLATES | Corning, USA |
|  | DMEM/F-12 MEDIA | Himedia, India |
|  | MTT | Himedia, India |
|  | DMSO | MP Biomedicals LLC, USA |
|  | BSA | Himedia, India |
|  | PVDF MEMBRANE | Thermo Fisher Scientific, USA |
|  | SAPONIN | Sigma, USA |
|  | DAPI | Himedia, India |
|  | VECTASHIELD® | Thermo Fisher Scientific, USA |
|  | CFSE | Thermo Fisher Scientific, USA |
|  | PROPIDIUM IODIDE (PI) | BD biosciences, USA |
|  | HBSS | Himedia, India |
|  | OCT medium | Thermo Fisher Scientific, USA |
|  | L-poly lysine | Sigma Aldrich, USA |
|  | Proparacaine hydrochloride ophthalmic solution | Sunways India Pvt Limited, India |
|  | Tropicamide ophthalmic solution | Sunways India Pvt Limited, India |
|  | Tobrex ointment | Alcon manufacture, India |
|  | Povidine-iodine | Cipladine, India |
|  | b FGF | Prospects Immunotools, Germany |
|  | EGF | Prospects Immunotools, Germany |
|  | Retinoic Acid | Sigma, USA |
|  | SCF | Prospects Immunotools, Germany |
|  | Insulin growth factor (IGF) – 1 | Prospects Immunotools, Germany |
|  | Taurine | Prospects Immunotools, Germany |
|  | Minimum Essential Medium – Non-Essential Amino Acids (MEM – NEAA) | Himedia, India |
|  | B-27 supplement | Sigma, USA |
|  | ESC grade serum | Himedia, India |
|  | IL3 | Prospects Immunotools, Germany |
|  | MCSF | Prospects Immunotools, Germany |
|  | 2-ME | Himedia, India |
|  | Hisep ficoll | (HiMedia, India;1.077 g/cm^3^) |
|  | Antibiotic | Himedia, India |
|  | Annexin-PI Staining kit | BD Biosciences, USA |
|  | Fibronectin | Prospects Immunotools, Germany |
|  | Trizol | TRI Reagent Sigma, USA |
|  | DEPC water | Himedia, India |
|  | SYBR green | Thermo Fisher Scientific,USA |
|  | cDNA synthesis Kit | Biorad,USA |
|  | Ethanol | Himedia, India |
|  | Fix/permeabilization solution | BD Biosciences, USA |
|  | Click-it EDU Alexa Flour 488 Assay kit | Thermo fisher Scientific,USA |
|  | BCA Kit | G-Biosciences,USA |
|  | SDS | Himedia, India |
|  | Polyacrylamide | Himedia, India |
|  | ECL Substrate Mixture | Thermo fisher scientific,USA |
|  | PFA | Himedia, India |
|  | OCT medium | Leica Biosystems |
|  | DetectX® cGMP colorimetric kit | Arbor Assays, USA, Cat: K020-H1 |
|  | DiBAC4 | Thermo fisher scientific,USA |
|  | Pluronic F127 | Thermo fisher scientific,USA |
|  | Valinomycin | Thermo fisher scientific,USA |
|  | Gramicidin | Thermo fisher scientific,USA |

**Supplementary Fig 1:** Monocytes isolated from PBMCs were stained for anti-human retinal marker intra-cellular Pax6 tagged with PE. The monocytes were run in BD FACSVerse^TM^. Panel A shows the FSC-SSC plot for PBMCs and the monocyte population was gated as Population of interest (P1). In Panel B, we applied the gate P1 to obtain only monocytes and analyzed for PAX-6 positive population. We did not find PAX-6 positive population in monocytes.


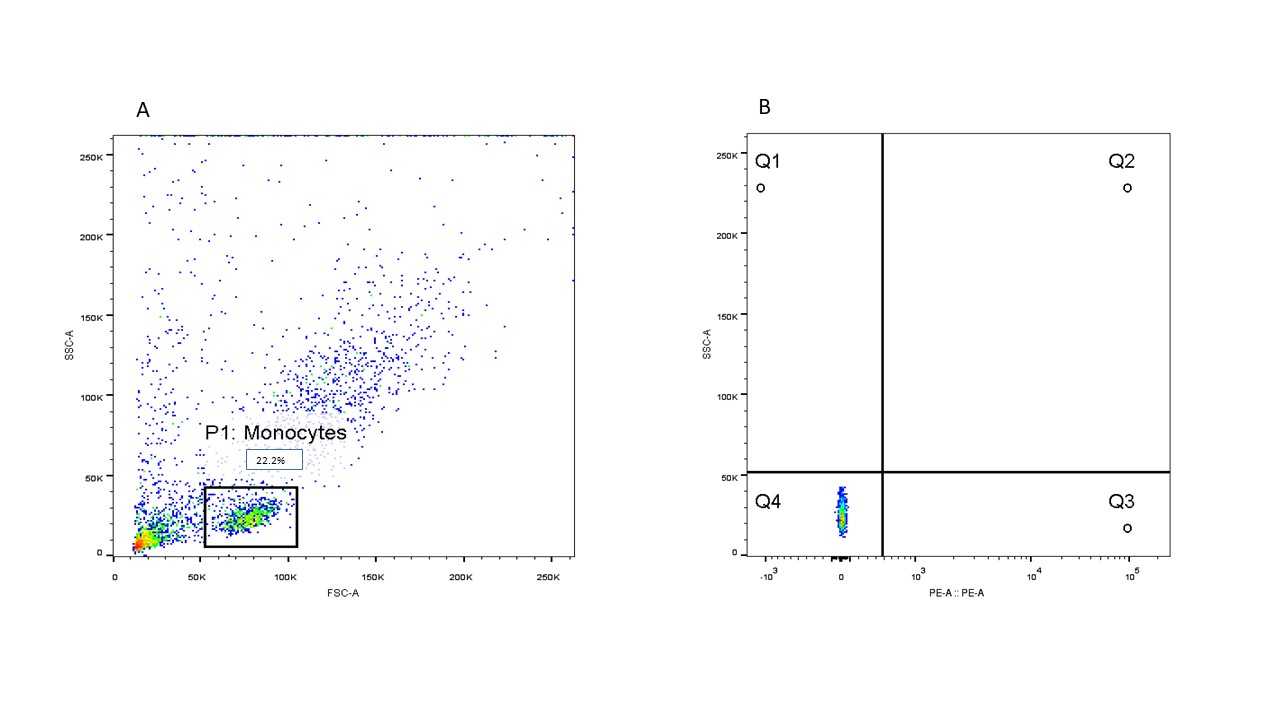


**Supplementary Fig 2**: Expression of neuronal markers on retinal neuron like cells (RNLCs). Monocytes were de-differentiated and induced with retinal growth factors. Majority of RNLCs expressed retinal and neuronal markers like Rhodopsin (Green) and Pax-6 (Red).

Rhodopsin on RNLCs Pax-6 on RNLCs


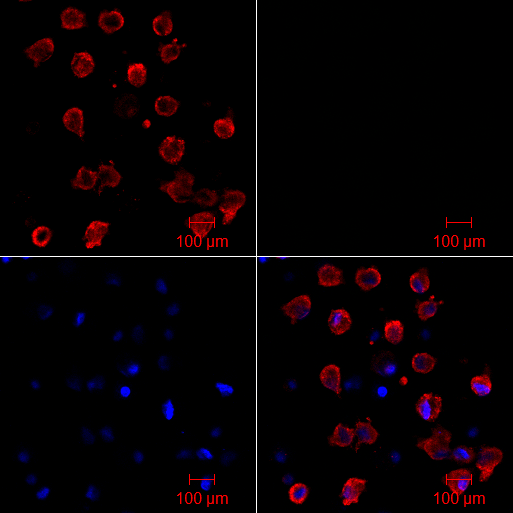

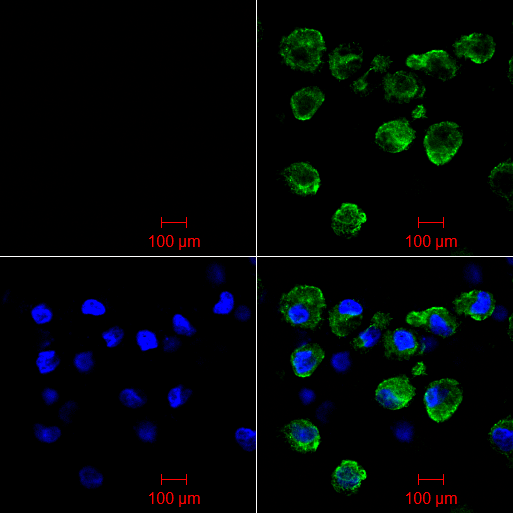


Rhodopsin on Monocytes Pax-6 on Monocytes

DAPI Rhodopsin Merge DAPI Pax-6 Merge


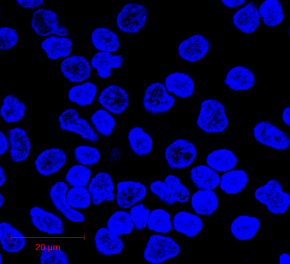

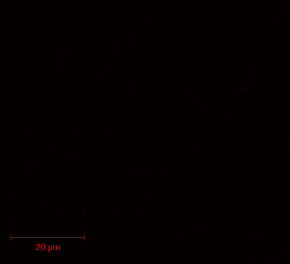

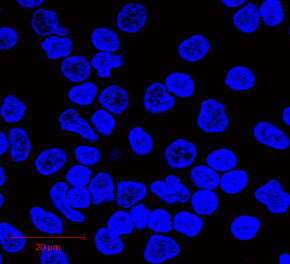

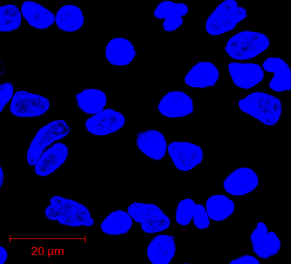

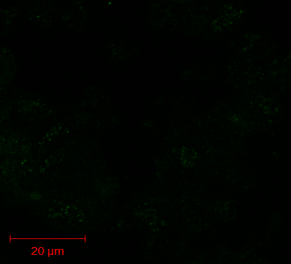

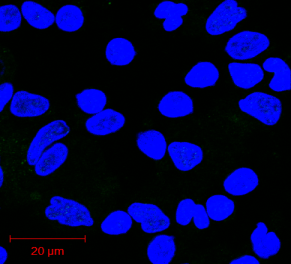


**Supplementary Fig 3:** One million monocytes were transplanted in the subretinal layer of the right eye in the NOD.SCID-rd1 mice. The contralateral eye was used as non-injected negative control. The eye was enucleated and stained with anti-human retinal makers *S-OPSIN, RCVRN and PDE6b* after 10 days. We did not find neuronal/retinal marker expression in retina upon monocyte transplantation, suggesting that monocytes failed to integrate or differentiate in vivo. Blue: DAPI, Fluorochrome for S-OPSIN, RCVRN and PDE6b was Alexa Fluor 488.


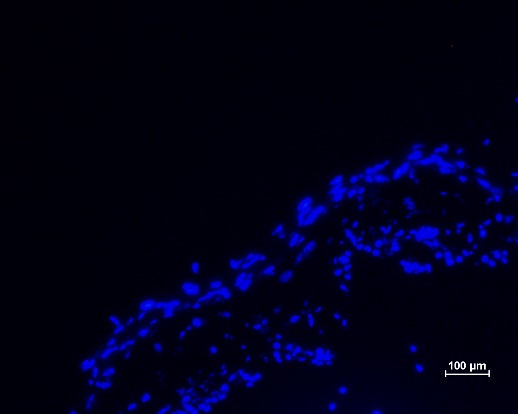

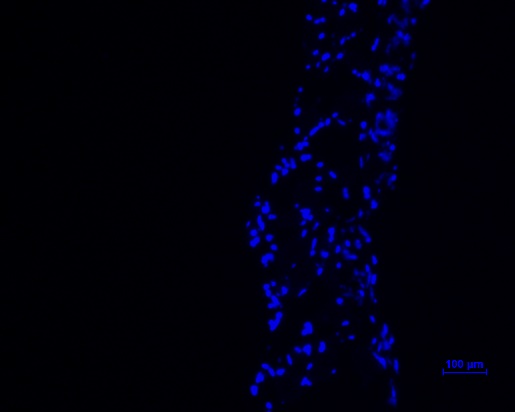

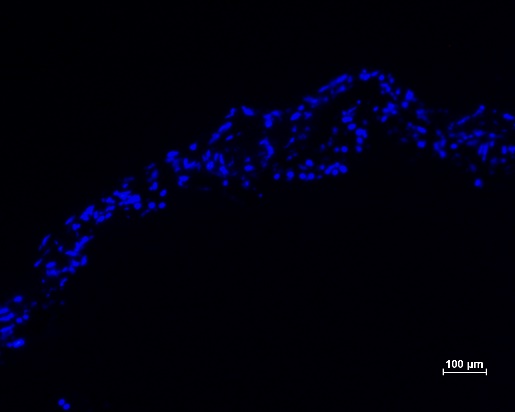

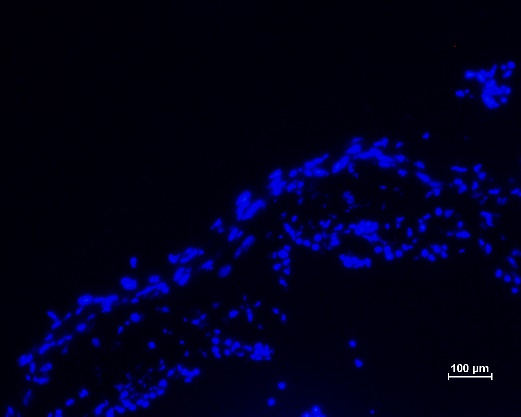

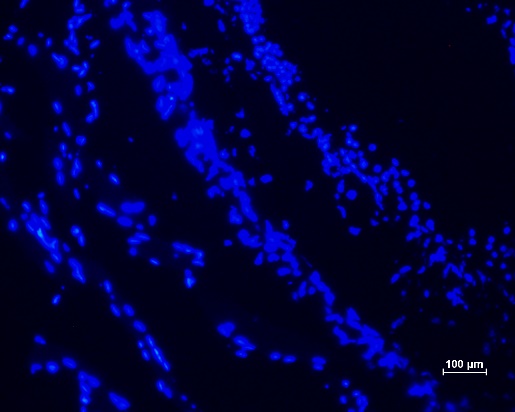

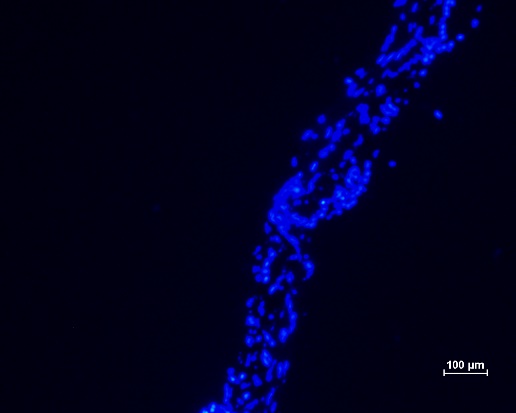


PDE6b RCVRN S-OPSIN

Non- Transplanted Control Monocyte Transplanted Control

RPE

RPE

RPE

RPE

RPE

RPE

**Supplementary Fig 4:** Paracrine signaling molecules were quantified by fluorometric analysis in vitro. For each culture time point (supernatant at day 3 and day 6 was collected in de-differentiation culture while day 10 and day 14 was from RNLC culture), media supernatant was collected separately from 10 different cultures and the analyte was quantified. (A) L-Glutamate (B) Acetylcholine (C) Nitric Oxide (n=10). ** = p<0.001; *** = p<0.0001. All these signaling molecules play a dual role as neurotransmitters and paracrine neuroprotectors during stress conditions in retina.

(A) (B)


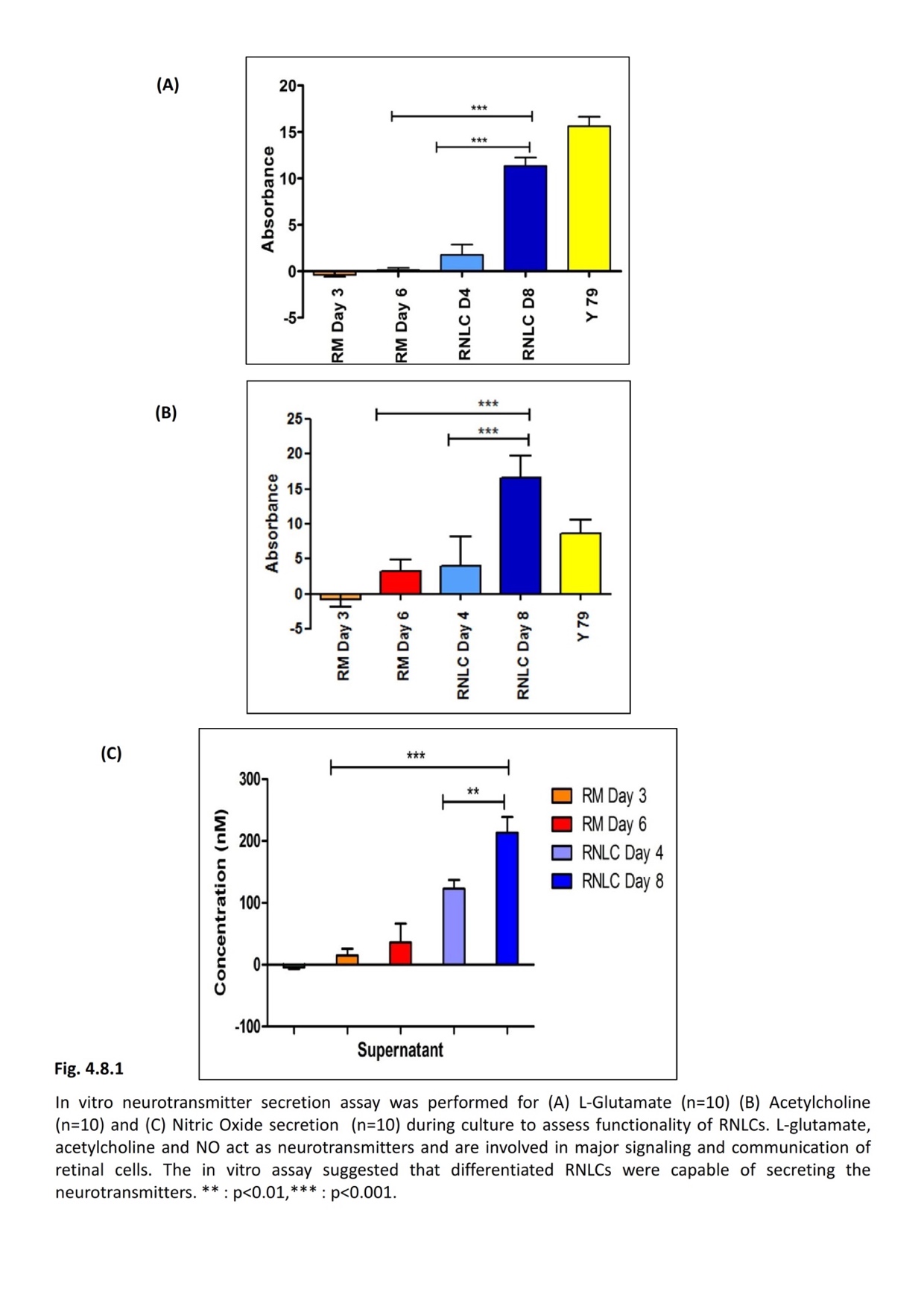


Day 3

Day 6

Day 10

Day 14

Y-79

Absorbance


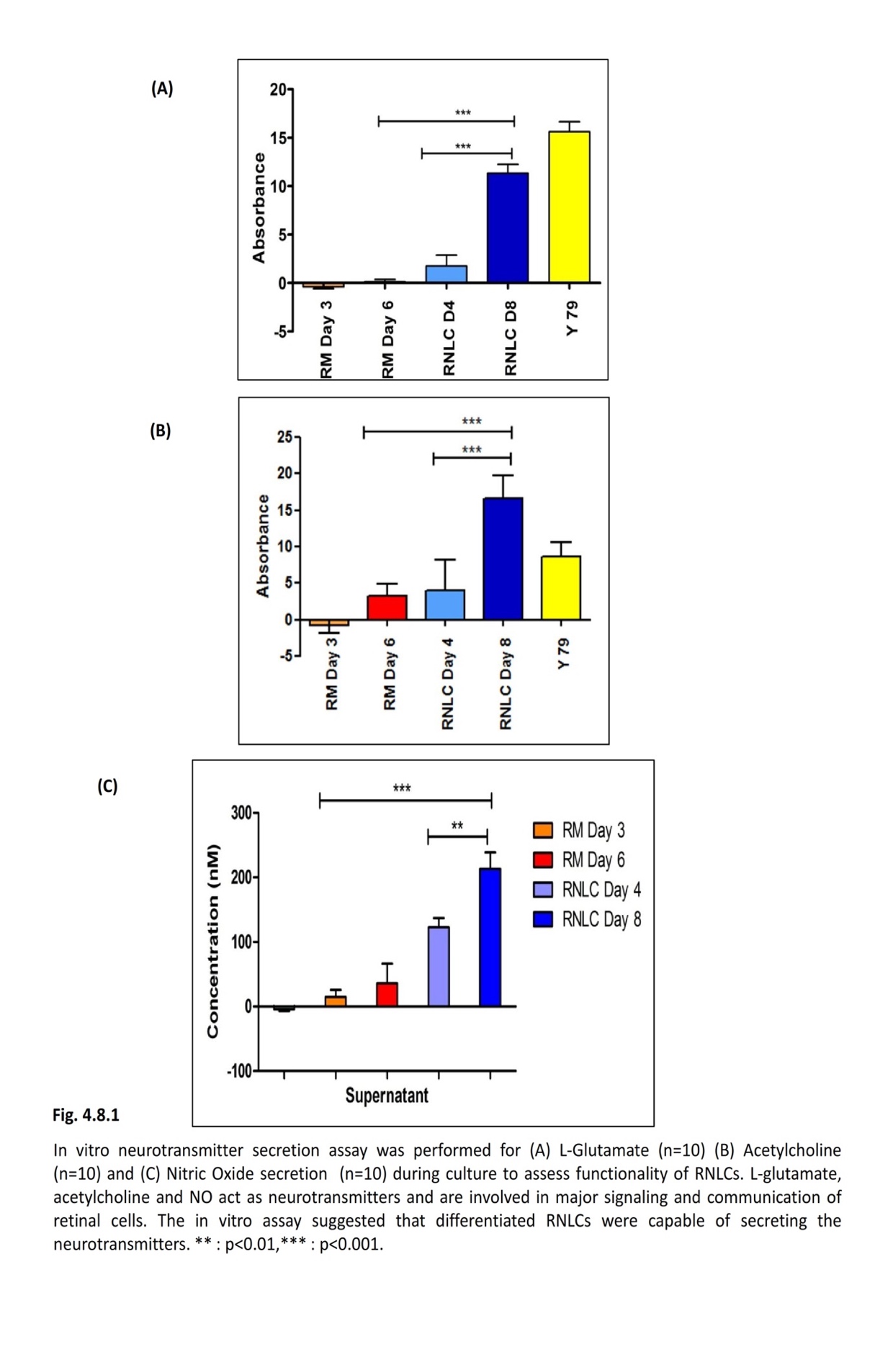


Day 3

Day 6

Day 10

Day 14

Y-79

Absorbance

(C)


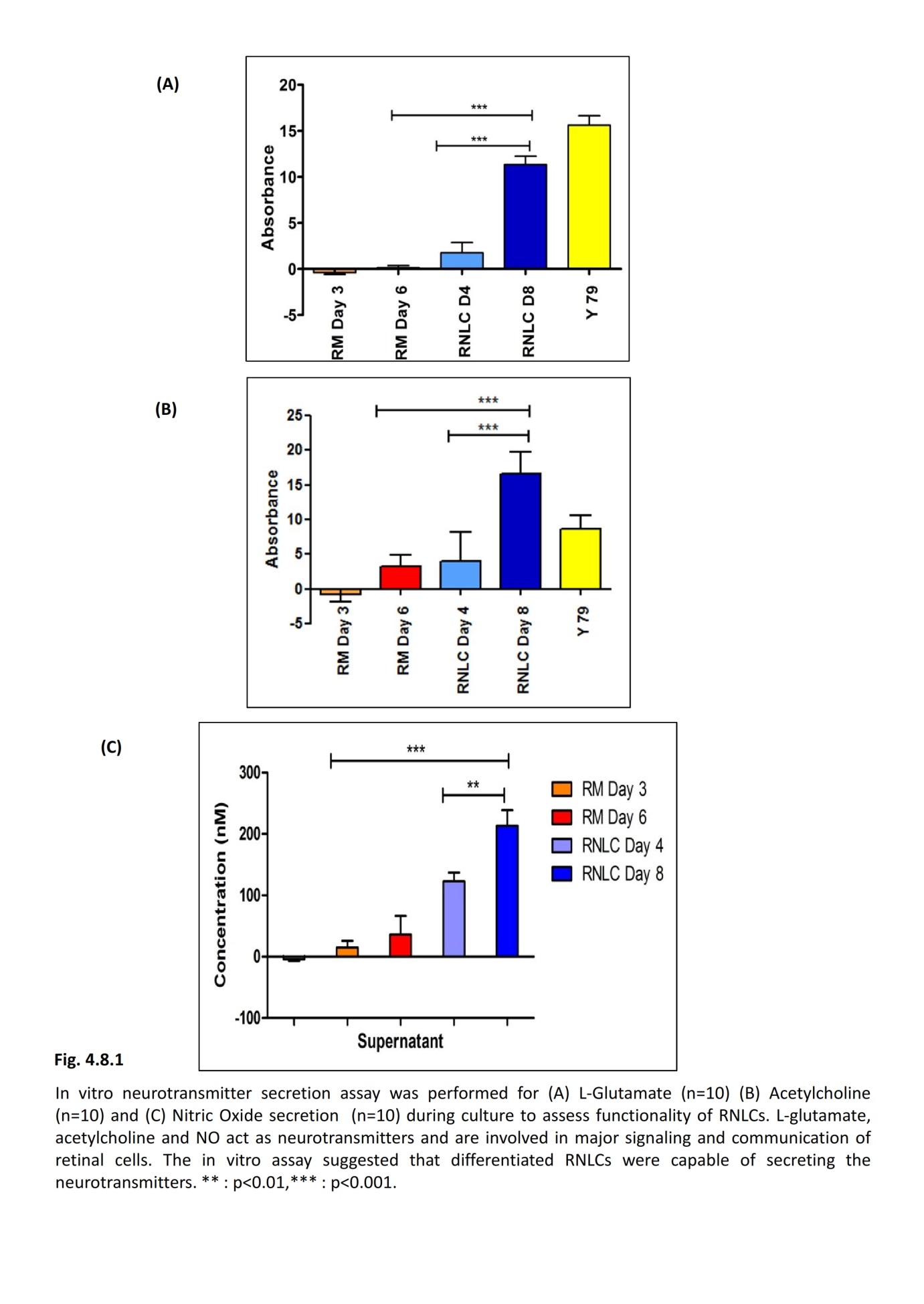

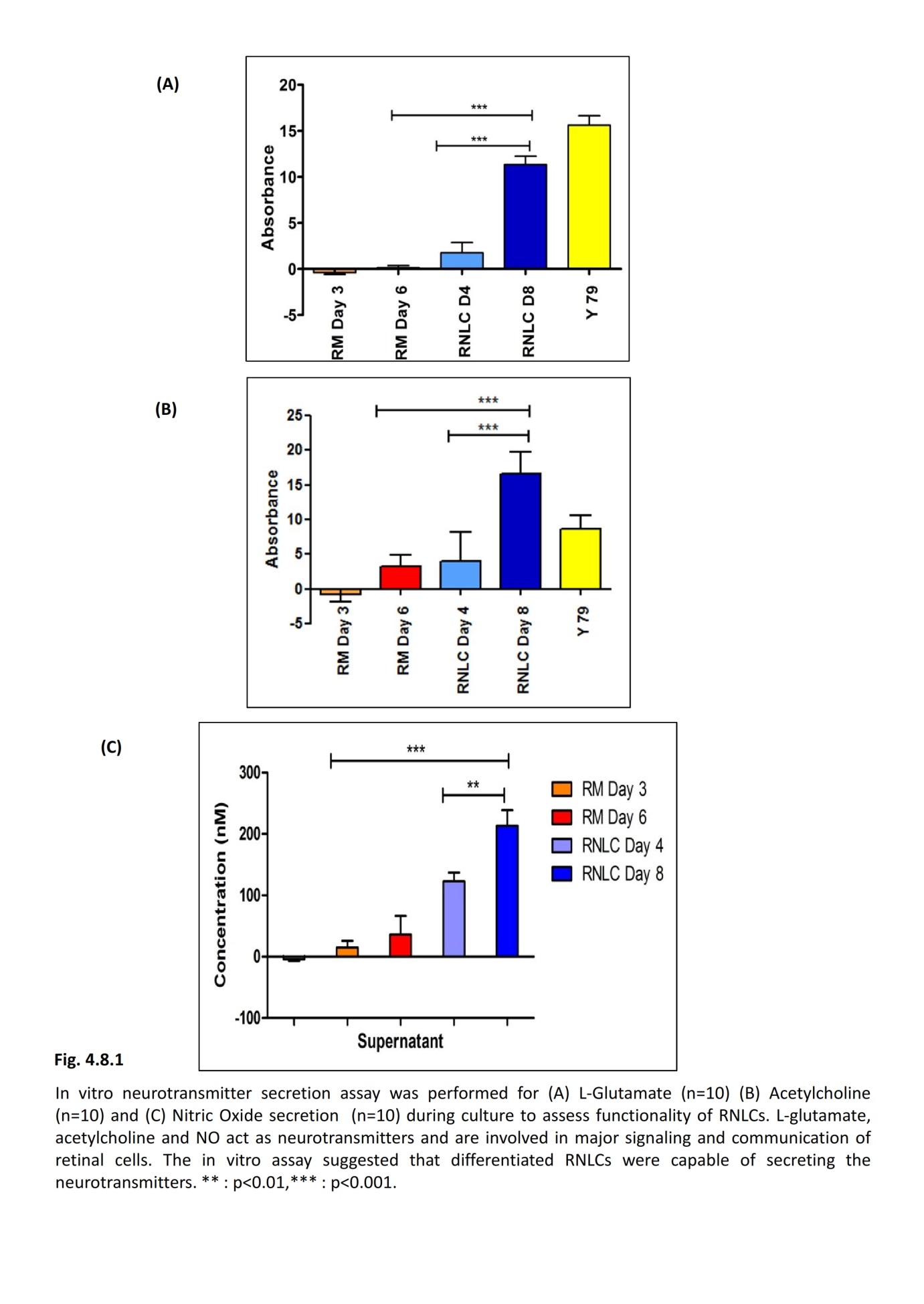


Day 3

Day 6

Day 10

Day 14
